# Dynamic Tripartite Governance in data security: An evolutionary game model with cross-level government supervision

**DOI:** 10.1371/journal.pone.0325473

**Published:** 2025-06-03

**Authors:** Shiyu Wang, Kai Zou, Yuxuan Zou, Zhiyi Jiang

**Affiliations:** 1 College of Public Administration, Xiangtan University, Xiangtan, China; 2 School of Management, Xi’an Jiaotong University, Xi’an, China; 3 Library of Xiangtan University, Xiangtan, China; Aalto University, FINLAND

## Abstract

As digital governance transitions to a decentralized architecture, data security has become a key driver of institutional modernization. This study constructs a three-party evolutionary game framework, integrating the grassroots government (GRG), local government (LG), and third-party regulator (TPR), and empirically calibrates the parameters using financial records from municipal data projects. Using replicator dynamics and Lyapunov stability analysis, we identify three key mechanisms in cross-level governance: 1. While reward systems enhance GRG compliance, excessive rewards, due to resource misallocation, weaken LG’s regulatory rigor. 2. When the overall penalties systematically exceed opportunistic gains, rent-seeking behavior is effectively suppressed, reducing the incentives for collusion between GRG and TPR. 3. Increasing the rent-seeking costs of TPR and enhancing social accountability benefits promote GRG’s sustained commitment to data security supervision. Simulations conducted in MATLAB illustrate the nonlinear interactions between governance parameters and reveal that dynamic reward and punishment mechanisms can accelerate convergence towards a stable regulatory equilibrium. This study proposes optimizing China’s grassroots data governance framework through dynamically adjusted reward and penalty mechanisms and increased rent-seeking costs, providing practical guidance for enhancing regulatory effectiveness. **Index Terms** data security governance, evolutionary game theory, incentive mechanisms, grassroots regulation, rent-seeking.

## Introduction

The Digital China Initiative was officially introduced in the “14th Five-Year Plan for the Development of Digital Economy” by the State Council, and it has become a strategic driving force for China’s modernization development model [1]. Under this framework, data, as a core production factor, has fundamentally reshaped the economic value creation mechanism [2]. The construction of the digital ecosystem is accompanied by the generation of vast amounts of data, while data security issues have also emerged as an urgent challenge that needs to be addressed [3]. According to the Security Insider, AT&T, a U.S. telecommunications company, suffered a data breach that exposed 73 million customer records, including personal information such as names, phone numbers, and mailing addresses [4]. The Wall Street Journal reported that the Shanghai Municipal Public Security Bureau’s database had been hacked, leaking personal information of over 1 billion residents of mainland China [5]. Unrestricted data collection, data analysis, data misuse, and hacking attacks are gradually threatening individual privacy, corporate security, and social stability. As a key tool to address data security issues, data security governance directly impacts national security and public interests. By incorporating security regulatory mechanisms into organizational structures, it effectively enhances organizational security capabilities and promotes coordination between data security and risk management [6,7].

China’s data security governance started relatively late, and the government still faces many challenges in regulating data practices across various industries. Strengthening regulatory capabilities and effectiveness is a crucial measure to ensure data security. As public concern over data security intensifies, academia is continuously updating its research to address new challenges. Current research primarily focuses on the interactions between the government and various industries [8,9]. However, the behavior of GRG, as a key player in data governance, has not received sufficient attention or in-depth discussion. The political changes at the grassroots level in 20th-century China were centered around the decentralization of national power. As a grassroots organization, township governments are tasked with enhancing grassroots social management and services, shifting the focus of social governance downward to increase the public’s sense of access, well-being, and security [10]. The promotion of digital government has prompted GRG to accelerate its data security governance efforts. GRG data security governance involves multiple participants, whose interactions form a dynamic game. As the frontline implementers of data security governance, GRG is directly responsible for the collection, processing, and compliance oversight of data. LG is responsible for the broad management of the regulatory framework, resource allocation, and penalties for non-compliant behavior. TPR provides regulatory services, ensuring the effective implementation of data security governance measures and helping the government identify potential risks through audit reports.

Therefore, this study constructs a dynamic evolutionary game model of cross-level government to analyze the interactive relationships between GRG, LG, and TPR in data security regulation, filling the gap in research on grassroots government data regulation. The model also incorporates rent-seeking behavior to explore its potential impact on data security regulation. Through the analysis, the model reveals how increasing the costs of rent-seeking can improve governance effectiveness. This provides new insights for optimizing the existing regulatory framework, particularly in terms of coordinating cooperation between various levels of government and TPR, offering new perspectives and theoretical support for data security governance, with significant academic value and practical significance.

## Background

The American National Standards Institute (ANSI) defines data governance as the process of managing data availability, security, and privacy within an enterprise, covering data lifecycle management [11]. The European Union, through modernized regulatory mechanisms, has set a broad territorial jurisdiction for data governance, enhancing its extraterritorial effectiveness [12]. Early research laid the foundation for data governance practices through comparative legal analysis and the construction of cross-national policy evaluation systems [13]. Subsequent studies introduced lifecycle theory, mapping security protocols for the stages of data collection, storage, and transmission in detail, while the pyramid of needs model further refined these frameworks by aligning technological safeguards with organizational priorities [14,15]. The open government data movement has driven scholars to deconstruct trade-offs in security risks, proposing evaluation matrices applicable to public platforms [16–18]. Factor analysis methods later identified key determinants of governance efficiency, such as encryption standards, access control, and audit frequency, providing actionable standards for institutional design [19,20]. In recent years, with the growing interest in adaptive regulation, research has shifted to focus on how to balance innovation and risk control in a dynamic regulatory environment. Europe’s data governance model has had a significant impact on China’s mixed approach to sovereignty protection and cross-border interoperability [21–26]. The EU’s General Data Protection Regulation (GDPR) promotes enforcement of cross-border data cases through a “one-stop” regulatory mechanism, ensuring shared authority between regulators, though it still faces challenges such as procedural opacity and inefficiency [27]. Therefore, evolutionary game theory has provided important theoretical support for multi-center governance, especially through risk regulation in public-private partnerships [28,29], hierarchical supervision under central-local execution strategies [30–34], and quality control mechanisms aligning incentives between producers and consumers in the data market [35–37]. Despite many studies recognizing the reward-punishment mechanism as a key lever in governance [38–41], their limitations fail to fully reveal the complexity of the governance process. Firstly, these studies often simplify government actors into a single entity, ignoring the diversity and conflicts of interest within the government [42–44]; secondly, static equilibrium assumptions cannot effectively explain the path-dependent nature of decentralized governance systems [45,46]; finally, they do not consider the potential rent-seeking behavior between game participants, which could lead to improper exchanges of benefits and exacerbate the risk of governance failure [47,48]. These limitations mean that in understanding and designing cross-level government supervision, the crucial role of grassroots governments in coordinating multiple interests and the challenges they face in implementation are often overlooked [49], especially in balancing interests between different levels in the face of rent-seeking behavior. The current literature on this type of cross-level governance is still insufficient, and how to effectively integrate relationships between levels and address data security issues remains an underexplored area.

Existing research provides theoretical frameworks for data security governance, but the role of GRG in multi-center governance within the three-party regulatory framework has not been deeply analyzed. Additionally, the literature on cross-level government supervision is sparse, failing to fully reveal the complex interactions between GRG, LG, and TPR. These research gaps leave us with insufficient understanding of the practical effects and potential issues of multi-party collaboration in data security governance. To address these issues, this paper proposes a game model that introduces rent-seeking behavior, combining the dynamic interactions between GRG, LG, and TPR, with the aim of filling this research gap, exploring the application of cross-level government supervision in data security governance, and offering new perspectives and solutions for improving the data security governance system.

### Evolutionary game model

#### Problem statement.

The continued occurrence of data security issues highlights the urgent need for government agencies to strengthen data security governance. These issues not only damage the government’s credibility but also have a significant impact on the public’s daily life. TPR, as a neutral entity, effectively complements the hierarchical supervision framework while increasing the credibility of GRG’s governance. GRG plays an execution and supervision role in data governance, but due to limitations in funding, technology, and personnel, it often lacks sufficient capabilities. TPR, on the other hand, is tasked with data compliance checks, privacy protection, and security audits. The professionalism and independence of these agencies make them valuable partners for both the government and businesses. In this context, rent-seeking behavior that may emerge between GRG and TPR has gradually become an undeniable phenomenon [50,51]. When GRG faces a lack of resources in data governance, the governance costs often exceed the losses that would occur in the short term by not conducting governance. Therefore, some GRGs may choose to cooperate with third-party regulatory agencies to avoid high governance costs. This rent-seeking behavior typically manifests as GRG choosing not to conduct data security governance, instead obtaining partially incomplete or substandard governance reports through rent-seeking, thereby reducing governance costs in the short term. TPR, driven by economic interests, refrains from regulation and either accepts or actively engages in rent-seeking behavior in exchange for benefits from GRG. In the process of providing compliance certifications, TPR may lower standards, delay the issuance of reports, or weaken the regulatory intensity in other ways. This behavior not only undermines the quality of data security governance but may also create hidden risks for data breaches, misuse, and other threats. Furthermore, the regulatory pressure on LG may prompt them to relax data security requirements to save costs or avoid penalties. This lack of regulation exacerbates governance failure, affecting public trust and data security.

**Fig 1:**
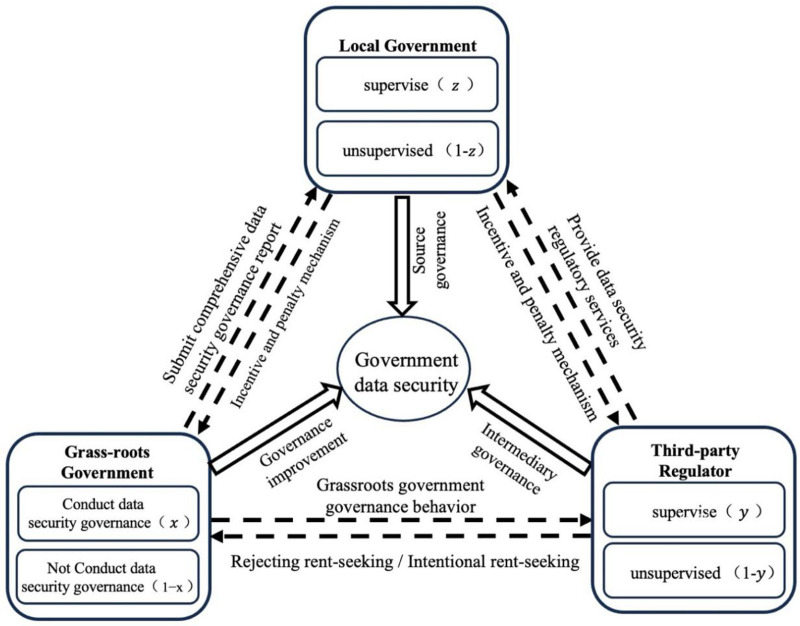
Game-behavior relationship of data security supervision in GRG.

Therefore, unlike previous regulatory models, the game model introduces an analysis of rent-seeking behavior, revealing the interest game mechanism between GRG and TPR. By simulating the impact of rent-seeking behavior, this approach helps policymakers identify potential issues in governance and design more effective regulatory strategies, ensuring the transparency and effectiveness of data governance. This research holds significant theoretical value and practical implications for government public governance in the current digital transformation. Fig 1 illustrates the tripartite game behavior in data security supervision within GRG.

### Model assumptions

In the context of GRG data security governance, we identify three bounded rational agents [52]: the grassroots government (GRG), the local government (LG), and third-party regulators (TPR). Each agent strategically adapts their choices based on others’ decisions within an evolutionary game framework. Key assumptions are grounded in prior studies on regulatory dynamics [53], incentive design [54], and multi-level governance [55].

**Assumption 1**: Let x∈ [0,1] represent the probability that GRG chooses to perform data security governance, and 1 − x represent the probability of choosing not to perform data security governance. Similarly, y∈ [0,1] and z∈ [0,1] represent the regulatory probabilities for TPR and LG, respectively.

**Assumption 2**: GRG performing data security governance generates a baseline profit $R_{p}$ while incurring governance costs $C_{z}$. If data governance is not performed, data security incidents may occur, leading to compensation and other costs, with this cost being $C_{b}$, where $C_{z}$>$C_{b}$. To avoid high governance costs, GRG may engage in rent-seeking with TPR to obtain a more comprehensive data security governance report, with the rent-seeking cost denoted as $B_{j}$, and $B_{j}$<($C_{z}$−$C_{b}$). Furthermore, speculative behavior from not performing data security governance will incur speculative costs, including lowering standards, delaying governance, and other management fees, with the speculative cost denoted as Cp.

**Assumption 3**: TPR chooses to supervise and earns a reward $V_{j}$. If TPR engages in rent-seeking with GRG and helps provide a more complete data governance report in exchange for benefits, TPR will bear the rent-seeking cost $C_{j}$.

**Assumption 4**: If GRG does not perform data security governance, LG will impose a penalty $F_{p}$, and if TPR chooses not to supervise, LG will impose a penalty $F_{j}$. Conversely, if GRG performs data security governance, it will receive a reward $M_{p}$, and if TPR chooses to supervise, it will receive an incentive $M_{j}$.

**Assumption 5**: LG’s choice to supervise incurs a cost $C_{d}$, and it gains a societal benefit $U_{d}$. If LG chooses not to supervise, the cost of rectifying the data security incident will be $Z_{d}$. A failure in regulation that causes a decline in government credibility will result in a penalty $T_{d}$ from the superior department, where $T_{d}$>$C_{d}$ to ensure incentive compatibility. When the cost $C_{d}$ is too high, LG may tolerate collusion between GRG and TPR to avoid bearing $T_{d}$, reflecting the real-world trade-offs within a hierarchical system.

The parameter definitions are provided in Table 1, which details the meaning of each parameter and its role and impact in the model. Through Table 1, readers can clearly see the specific values of the parameters used in the model and how they influence the model’s operation and outcomes.

**Table 1:** Parameter Settings.

Parameters	Meaning
x	GRG’s strategy choice
y	TPR’s strategy choice
z	LG’s strategy choice
$C_{z}$	Cost of GRG choosing to perform data security governance, such as upgrading protection systems, training management personnel, etc.
$C_{b}$	Cost of GRG choosing not to perform data security governance, such as compensation for losses, repair work, etc.
$C_{p}$	Speculative cost of GRG, such as emergency handling, lowering standards, etc.
$B_{j}$	Rent-seeking cost of GRG to TPR, such as obtaining a comprehensive report, reviewing results, etc.
$C_{j}$	Cost of TPR engaging in rent-seeking behavior, such as providing false reports, falsifying regulatory processes, etc.
$C_{d}$	Cost of LG choosing to supervise, such as technical costs, human and material resource costs, etc.
$Z_{d}$	Cost of LG choosing not to supervise, such as investigation fees, remediation costs, etc.
$R_{p}$	Original profit of GRG performing data security governance, such as enhanced government credibility, improved decision-making efficiency, etc.
$V_{j}$	Profit of TPR choosing to supervise, such as business expansion, establishing new partnerships, etc.
$U_{d}$	Social benefits obtained by LG, such as reputation enhancement and innovation performance, etc.
$M_{p}$	Reward given by LG to GRG for choosing to perform data security governance, such as subsidies, tax incentives, etc.
$M_{j}$	Reward given by LG to TPR for choosing to supervise, such as material rewards, honor recognition, etc.
$F_{j}$	Penalty imposed by LG on TPR for choosing not to supervise, such as confiscating illegal earnings, fines, etc.
$F_{p}$	Penalty imposed by LG on GRG for choosing not to perform data security governance, such as accountability, economic fines, etc.
$T_{d}$	Penalty imposed by the superior department on LG for choosing not to supervise, such as public reprimands, party and government disciplinary actions, etc.

#### Model construction.

According to the above assumptions, Table 2 shows the evolutionary game matrix of the tripartite subjects of the GRG, the TPR, and the LG.

**Table 2:** Evolutionary game matrix of GRG, TPR, and LG.

		Third-party Regulator	Local Government	
			Supervise z	Unsupervised 1−z
Grass-roots Government	Conduct data security governance x	Supervise y	$R_{p}-C_{z}+M_{p},V_{j}+M_{j},$ $-C_{d}-M_{p}-M_{j}+U_{d}$	$R_{p}-C_{z},V_{j},U_{d}$
		Unsupervised 1−y	$R_{p}-C_{z}+M_{p},V_{j}-C_{j}-F_{j},$ $-C_{d}-M_{p}+F_{j}+U_{d}$	$R_{p}-C_{z},V_{j}-C_{j},U_{d}$
	Not conduct data security governance 1−x	Supervise y	$-C_{b}-C_{p}-F_{p},Vj+M_{j},$ $-C_{d}+F_{p}-M_{j}$	$-C_{b}-C_{p},V_{j},0$
		Unsupervised 1−y	$R_{p}-C_{b}-C_{p}-B_{j}-F_{p},$ $V_{j}-C_{j}+B_{j}-Fj$ $-C_{p}+F_{p}+F_{j}-Z_{d}$	$R_{p}-C_{b}-C_{p}-B_{j}$ $Vj-C_{j}+B_{j},-Z_{d}-T_{d}$

### Replication dynamic equation

#### Strategic stability analysis of GRG.

With an expected benefit of E11 for a GRG that chooses to conduct data security governance, E12 for one that chooses not to conduct data security governance, and an average expected benefit of $\overset{―}{E1}$, Therefore:


$$\mathrm{\backslash [}{E11=yz\;[R}_{p}-C_{z}+M_{p}]+y(1-z)\;[R_{p}-C_{z}]+z(1-y)\;[R_{p}-C_{z}+M_{p}]+\mathrm{\backslash ]}$$

$$\mathrm{\backslash [}(1-y)(1-z)\;[R_{p}-C_{z}]\mathrm{\backslash ]}$$

$$=R_{p}-C_{z}+{zM}_{p}$$ (1)

$$\mathrm{\backslash [}E12=yz\left[-C_{b}-C_{p}-F_{p}\right]+y(1-z)\left[-C_{b}-C_{p}\right]+z(1-y)\left[R_{p}-C_{b}-C_{p}-B_{j}-F_{p}\right]+(1-y)(1-z)\left[R_{p}-C_{b}-C_{p}-B_{j}\right)\mathrm{\backslash ]}$$

$$=-C_{b}-C_{p}-zF_{p}+{y(B_{j}-R}_{p})+R_{p}-B_{j}$$ (2)

$$\overset{―}{E1}=xE11+(1-x)E12$$ (3)

The replication dynamic equation for the strategy choice of the GRG is derived from equations (1), (2), and(3) as follows:


$$\mathrm{\backslash [}F(x)=\frac{dx}{dt}=x\left(E11-\overset{―}{E1}\right)=x(1-x)[E11-E12]\mathrm{\backslash ]}$$

$$\mathrm{\backslash [}=x(x-1)\left[C_{z}-C_{b}-C_{p}-B_{j}-y\left(R_{p}-B_{j}\right)-z\left(F_{p}+M_{p}\right)\right]\mathrm{\backslash ]}$$ (4)

The first-order derivatives of x and the set G(y) are respectively:


$$\frac{d\left(F(x)\right)}{dx}=(2x-1[C_{z}-C_{b}-C_{p}-B_{j}-y(R_{p}-B_{j})-z(F_{p}+M_{p})$$ (5)

$$LetG(y)=C_{z}-C_{b}-C_{p}-B_{j}-y(R_{p}-B_{j})-z(F_{p}+M_{p})$$ (6)

According to the stability theorem of differential equations, the probability that the GRG will carry out data security governance in a steady state must be satisfied.

F(x)=0 and $\frac{d(F(x))}{dx}<0$. The solution is obtained from F(x)=0 for x=0, x=1, and G(y)=0. Since the first-order derivative of G(y)is less than 0, G(y) is a decreasing function on y.

G(y)=0 when $y=\frac{C_{z}-C_{b}-C_{p}-B_{j}-z(F_{p}+M_{p})}{R_{p}-B_{j}}=y^{*}$, at which point $\frac{d(F(x))}{dx}=0$ and the GRG is unable to determine an evolutionary stabilisation strategy;

When y<y*, G(y)>0, then ${\frac{d(F(x))}{dx}|}_{x=0}<0$, x=0 is the Evolutionarily Stable Strategy (ESS) of the GRG; conversely, x=1 is the ESS.

**Fig 2:**
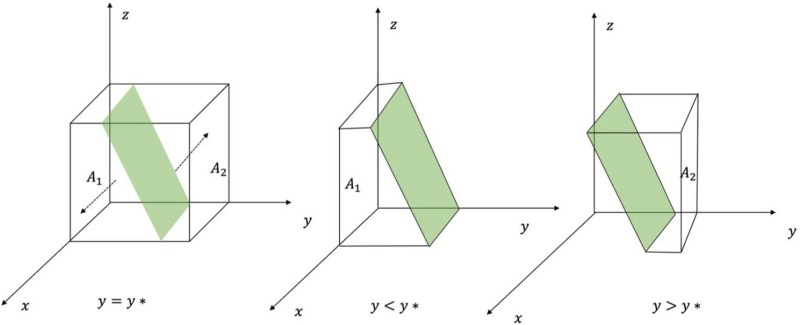
Phase diagram of the evolution of GRG strategies.

The phase diagram of the evolution of GRG strategies is shown in Fig 2.

From Fig 2, the volume of the probability $A_{1}$ of the GRG choosing not to carry out data security governance is $V_{A_{1}}$, and the volume of the probability $A_{2}$ of choosing to carry out data security governance is $V_{A_{2}}$, which is calculated:


$$\mathrm{\backslash [}V_{A_{1}}\text{=}\int_{\text{0}}^{\text{1}}\int_{\text{0}}^{\text{1}}\frac{C_{z}-C_{b}-C_{p}-B_{j}-z\left(F_{p}+M_{p}\right)}{\left(R_{p}-B_{j}\right)}\text{dzdx}\mathrm{\backslash ]}$$

$$=\frac{2\left(C_{z}-C_{b}-C_{p}-B_{j}\right)-(F_{p}+M_{p})}{2(R_{p}-B_{j})}$$ (7)

$$V_{A_{2}}=1-V_{A_{1}}$$ (8)

**Corollary 1** The probability that a GRG conducts data security governance is positively correlated with the raw benefits, breaches costs, supervisory costs, and rewards and penalties of the LG and negatively correlated with the cost savings of not conducting data security governance.

**Proof:** First-order partial derivatives are obtained for each element in $V_{A_{2}}$


$$\mathrm{\backslash [}\frac{\partial V_{A_{2}}}{\partial R_{p}}>0,\;\frac{\partial V_{A_{2}}}{\partial B_{j}}>0,\;\frac{\partial V_{A_{2}}}{\partial C_{p}}>0,\;\frac{\partial V_{A_{2}}}{\partial (C_{z}-C_{b})}<0\mathrm{\backslash ]}$$

From the first-order partial derivatives, an increase in $R_{p}$, $B_{j}$, ($F_{p}$+$M_{p}$), and $C_{p}$, as well as a decrease in ($C_{z}$-$C_{b}$), decreases $V_{A_{1}}$. It increases $V_{A_{2}}$, i.e., the probability that the GRG chooses to conduct data security governance increases.

**Corollary 1 shows that** the fundamental benefits of data security governance are crucial in deterring GRG from overlooking governance risks. LG can discourage neglect of data security governance among GRG through rewards and punishments and amplifying non-compliance costs. This can be done by enhancing influential tools such as expanding government open platforms, encouraging GRG to commit to data security governance.

**Corollary 2** The probability that a GRG conducts data security governance as the game evolves increases with the likelihood that the regulator refuses to provide rent-seeking behaviour and that the LG chooses to supervise

**Proof:** From the stability analysis of GRG governance strategy, when $z<C_{z}-C_{b}-C_{p}-B_{j}-\frac{y\left(R_{p}-B_{j}\right)}{F_{p}+M_{p}},y<y^{*},G(y)>0,$ at this time, ${\frac{d(F(x))}{dx}|}_{x=0}<0,$then x=0 that is, the GRG does not conduct data security governance as an evolutionarily stable strategy. Therefore, as the probability of TPR and LG choosing to supervise increases, the probability of GRG choosing to conduct data security governance increases from 0 to 1.

**Corollary 2 shows that** the probability that a GRG will engage in data security governance as a stabilization strategy increases with the probability that a TPR and a LG will choose to supervise. The process of LG regulation not only improves the probability of data security governance by GRG but also develops the social attributes of TPR. They are utilising media opinion to monitor the situation of data security governance at the grassroots level of government and provide wider channels for GRG data security supervision.

#### Strategic stability analysis of stability analysis of TPR.

The expected return for a TPR who chooses to supervise is E21, and the expected return for a TPR who chooses not to supervise and provide rentseeking behaviour, etc., is E22. The average expected return is $\overset{―}{E2}$ respectively


$$E21=x\left[z\left(V_{j}+M_{j}\right)+(1-z)V_{j}\right]+(1-x)\left[z\left(V_{j}+M_{j}\right)+(1-z)V_{j}\right]$$ (9)

$$\mathrm{\backslash [}E22=x\left[z\left(V_{j}-C_{j}-F_{j}\right)+(1-z)\left(V_{j}-C_{j}\right)\right]+(1-x)\left[z\left(V_{j}-C_{j}+B_{j}-F_{j}\right)+(1-z)\left(V_{j}-C_{j}+B_{j}\right)\right]\mathrm{\backslash ]}$$

$$\mathrm{\backslash [}=x\left(V_{j}-C_{j}-zF_{j}\right)+(1-x)\left(V_{j}-C_{j}+B_{j}-zF_{j}\right)\mathrm{\backslash ]}$$ (10)

$$\overset{―}{E2}=yE21+(1-y)E22$$ (11)

The replication dynamic equation for the TPR strategy choice is derived by solving the system of equations (9), (10), and (11) as follows:


$$\mathrm{\backslash [}F(y)=\frac{dy}{dt}=y(E21-\overset{―}{E2})=y(1-y)\;[E21-E22]\mathrm{\backslash ]}$$

$$=y(y-1[(1-x)B_{j}-C_{j}-z(F_{j}+M_{j})]$$ (12)

The first-order derivatives of y and the set G(z) are, respectively:


$$\mathrm{\backslash [}\frac{d(F(y))}{dy}=(2y-1)\left[(1-x)B_{j}-C_{j}-z\left(F_{j}+M_{j}\right)\right]\mathrm{\backslash ]}$$ (13)

$$LetG(z)=(1-x)B_{j}-C_{j}-z\left(F_{j}+M_{j}\right)$$ (14)

According to the stability principle of differential equations, the probability that the TPR chooses to supervise in a steady state must be satisfied:

F(y)=0 and $\frac{d(F(y))}{dy}$*<0*. The solution is obtained from F(y)=0 for y=0, y=1, and G(z)=0. Since the first-order derivative of G(z) is less than 0, G(z) is a decreasing function on z.

When $z=\frac{(1-x)B_{j}-C_{j}}{F_{j}+M_{j}}=z*$, G(z)=0, $\frac{d\left(F(y)\right)}{dy}=0$, and F(y)=0. At this point, the TPR is unable to determine a stabilization strategy;

When z<z*, G(z)>0 and ${\frac{d\left(F(y)\right)}{dy}|}_{y=0}<0$, then y=0 is the stable evolutionary strategy of the TPR; conversely, when z>z*, y=1 is the stable evolutionary strategy of the TPR.

From $z=z*=\frac{(1-x)B_{j}-C_{j}}{F_{j}+M_{j}}$, it follows that the coordinates of the two points on the phase diagram plane at the intersections of the x-axis and z-axis are $\left(\frac{B_{j}-C_{j}}{B_{j}},0,0\right)$ and $\left(0,0,\frac{B_{j}-C_{j}}{F_{j}+M_{j}}\right)$.

**Fig 3:**
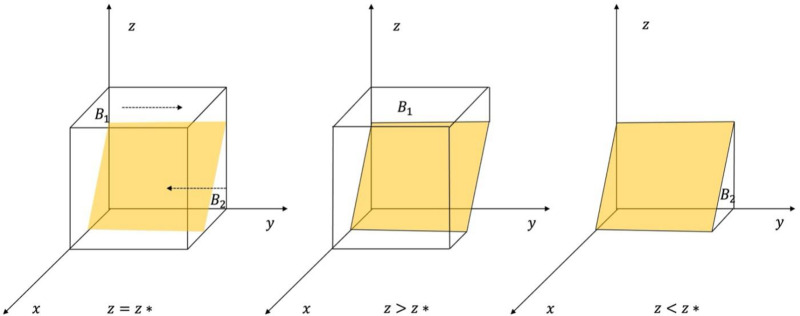
Phase diagram of the evolution of TPR’s strategies.

A phase diagram of the strategy evolution of TPR is shown in Fig 3.

From Fig 3, the probability that a third-party organization chooses to supervise is $B_{1}$ and the volume is $V_{B_{1}}$. The probability that it chooses not to supervise and provides rent-seeking behaviour for the LG is $B_{2}$, and the volume is $V_{B_{2}}$. calculated:


$$\mathrm{\backslash [}V_{B_{2}}=\int_{0}^{1}\int_{0}^{\frac{B_{j}-C_{j}}{B_{j}}}\frac{(1-x)B_{j}-C_{j}}{F_{j}+M_{j}}dxdy\mathrm{\backslash ]}$$

$$=\frac{{\left(B_{j}-C_{j}\right)}^{2}}{2\left(F_{j}+M_{j}\right)B_{j}}$$ (15)

$$V_{B_{1}}=1-V_{B_{2}}=1-\frac{{\left(B_{j}-C_{j}\right)}^{2}}{2\left(F_{j}+M_{j}\right)B_{j}}$$ (16)

**Corollary 3** The probability that a TPR chooses to supervise is negatively correlated with its benefits and positively correlated with the costs of behaviors such as rewarding LG for supervising, penalizing them for choosing not to supervise, and providing rent-seeking behavior to GRG.

**Proof:** The first-order partial derivatives of each element separately for $V_{B_{1}}$ yield: $\frac{\partial V_{B_{1}}}{\partial B_{j}}<0$, $\frac{\partial V_{B_{1}}}{\partial C_{j}}>0$, $\frac{\partial V_{B_{1}}}{\partial F_{j}}>0$, $\frac{\partial V_{B_{1}}}{\partial M_{j}}>0$. Therefore, when the rent-seeking costs for the GRG to obtain TPR decrease, and when the government increases the rewards and penalties for third-party institutions, as well as when TPR raises the rent-seeking costs, the probability of the third-party regulatory institutions choosing to regulate will increase.

**Corollary 3 shows that** when TPR benefit significantly from overseeing GRG and engaging in rent-seeking behaviors, LG must intensify their monitoring of these regulators. At the same time, efforts should be made to enhance the professional training of TPR staff and strengthen public participation in supervision, among other measures, in order to increase the costs of rent-seeking behavior provided by TPR. This will help promote fair and impartial regulation of local government data security governance by TPR.

**Corollary 4** The probability that a TPR chooses to supervise increases with the probability that a LG and a GRG choose to conduct data security.

**Proof:** When z<z* and $x<B_{j}-M_{j}-C_{j}-z(F_{j}+M_{j})/(B_{j}-M_{j})=x*$, y=0 is an evolutionarily stable strategy, and conversely when z>z* and x>x*, y=1 is an evolutionarily stable strategy. Therefore, it can be seen that when the probability of the GRG choosing data security governance and the LG choosing to supervise increases, the probability of the TPR choosing to supervise also increases from 0 to 1.

**Corollary 4 shows that** TPR’s strategic choices vary with the grassroots and LG’s supervisory strategies. The choice of r supervision by LG and the increased investment in data security governance by GRG all contribute to the increased probability of a TPR choosing to supervise as a stabilization strategy. Therefore, to ensure that data security governance is carried out orderly, LG should put forward strict supervisory requirements for TPR, provide specific incentives and support to GRG that conduct data security governance, and cultivate GRG’s awareness of data governance.

#### Strategic stability analysis of LG.

With a return of E31 for LG choosing to supervise, an expected return of E32for not supervising and an average expected return of $\overset{―}{E3}$*,* there is:


$$\mathrm{\backslash [}E31=xy\left(-C_{d}-M_{p}-M_{j}+U_{d}\right)+x(1-y)\left(-C_{d}-M_{p}+F_{j}+U_{d}\right)+y(1-x)\left(-C_{d}+F_{p}-M_{j}\right)+(1-x)(1-y)\left(-C_{p}+F_{p}+F_{j}-Z_{d}\right)\mathrm{\backslash ]}$$

$$=-C_{d}+xU_{d}-xM_{p}-yM_{j}+(1-x)F_{p}+(1-y)F_{j}-(1-x)(1-y)Z_{d}$$ (17)

$$\mathrm{\backslash [}E32=xy\left(U_{d}\right)+x(1-y)\left(U_{d}\right)+(1-x)(1-y)\left(-Z_{d}-T_{d}\right)\mathrm{\backslash ]}$$

$$=xU_{d}+(1-x)(1-y)(-Z_{d}-T_{d})$$ (18)

$$\overset{―}{E3}=zE31+(1-z)E32$$ (19)

The replication dynamic equation for the LG strategy choice is derived by solving the system of equations (17), (18), and (19) as follows:


$$\mathrm{\backslash [}F(z)=\frac{dz}{dt}=z(E31-\overset{―}{E3})=z(1-z)\;[E31-E32]\mathrm{\backslash ]}$$

$$=z(z-1[x(F_{p}+M_{p}+T_{d})-xyT_{d}+C_{d}-F_{j}-F_{p}-T_{d}+y(F_{j}+M_{j}+T_{d})]$$ (20)

The first-order derivatives of z and the set H(y) are respectively:


$$\mathrm{\backslash [}\frac{dF(z)}{dz}=(2y-1)\left[x\left(F_{p}+M_{p}+T_{d}\right)-xyT_{d}+C_{d}-F_{j}-F_{p}-T_{d}+y\left(F_{j}+M_{j}+T_{d}\right)\right]\mathrm{\backslash ]}$$ (21)

$$LetH(y)=x\left(F_{p}+M_{p}+T_{d}\right)-xyT_{d}+C_{d}-F_{j}-F_{p}-T_{d}+y\left(F_{j}+M_{j}+T_{d}\right)$$ (22)

According to the stability principle of differential equations, the probability that the LG chooses to supervise is steady must be satisfied: F(z)=0 and $\frac{d(F(z))}{dz}<0$. Since $\frac{\partial H(y)}{\partial y}$>0, i.e., H(y) is an increasing function concerning y.

When$y=\frac{F_{j}+F_{p}+T_{d}-C_{d}-x(F_{p}+M_{p}+T_{d})}{F_{j}+M_{j}+T_{d}-xT_{d}}=y**$, H(y)=0, $\frac{d(F(z))}{dz}=0$, at this point the LG cannot determine a stabilization strategy;

When y<y**, H(y)<0, ${\frac{d(F(z))}{dz}|}_{z=1}<0$, z=1 is the stabilizing evolutionary strategy of the LG; Conversely, z=0 is a stable evolutionary strategy for LG when y>y**.

**Fig 4:**
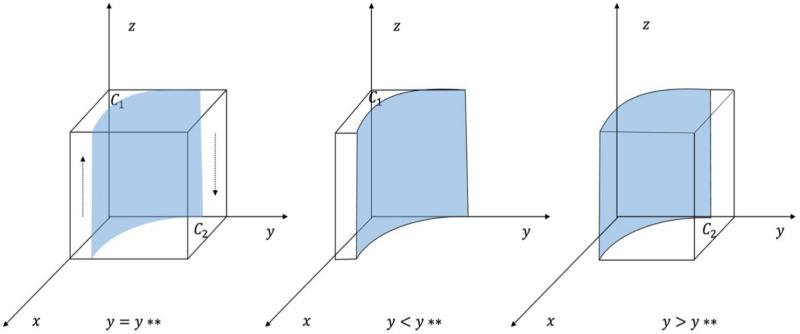
Phase diagram of LG strategy evolution.

A phase diagram of the evolution of LG strategies is shown in Fig 4.

From Fig 4, the probability that the LG chooses to supervise is $C_{1}$, with volume $V_{C_{1}}$, and it chooses not to supervise is $C_{2}$, with volume $V_{C_{2}}$. Calculated:


$$\mathrm{\backslash [}V_{C_{1}}=\int_{0}^{1}\int_{0}^{1}\frac{F_{j}+F_{p}+T_{d}-C_{d}-x\left(F_{p}+M_{p}+T_{d}\right)}{F_{j}+M_{j}+T_{d}-xT_{d}}dxdz\mathrm{\backslash ]}$$

$$=\frac{F_{p}+M_{p}+T_{d}}{T_{d}}-\left[\frac{M_{p}+M_{j}+C_{d}}{T_{d}}+\frac{(F_{p}+M_{p})(F_{j}+M_{j})}{{T_{d}}^{2}}\right]\ln\left(1+\frac{T_{d}}{M_{j}+F_{j}}\right)$$ (22)

$$V_{C_{2}}=\left[\frac{M_{p}+M_{j}+C_{d}}{T_{d}}+\frac{(F_{p}+M_{p})(F_{j}+M_{j})}{{T_{d}}^{2}}\right]\ln\left(1+\frac{T_{d}}{M_{j}+F_{j}}\right)-\frac{M_{p}+F_{p}}{T_{d}}$$ (23)

**Corollary 5** The probability that a LG chooses to supervise increases with an increase in LG fines to the GRG and administrative penalties from higher levels and decreases with an increase in LG incentives to the GRG and TPR.

**Proof:** The first-order partial derivatives of each element of $V_{C_{1}}$ are obtained as $1-\frac{M_{j}+F_{j}}{F_{j}+M_{j}+T_{d}}$*<*$\ln\left(1+\frac{T_{d}}{M_{j}+F_{j}}\right)$*<*$\frac{T_{d}}{M_{j}+F_{j}}$, and we obtain: $\frac{\partial V_{C_{1}}}{\partial F_{j}}>0$(($M_{p}$+$M_{j}$-$C_{d}$)-($M_{j}$+$F_{j}$+$T_{d}$)>0), $\frac{\partial V_{C_{1}}}{\partial F_{p}}>0$, $\frac{\partial V_{C_{1}}}{\partial M_{p}}<0$, $\frac{\partial V_{C_{1}}}{\partial M_{j}}<0$, $\frac{\partial V_{C_{1}}}{\partial T_{d}}>0$. Therefore, when ($M_{p}$+$M_{j}$-$C_{d}$)-($M_{j}$+$F_{j}$+$T_{d}$)>0, an increase in $F_{j}$ increases $V_{C_{1}}$, i.e., the probability that the LG chooses to supervise increases. From the above partial derivatives, it is clear that an increase in the amount of LG penalties and a decrease in the amount of incentives increases the probability that the LG chooses to supervise.

**Corollary 5 shows that** LG’s probability of supervising is related to the incentives and disincentives mechanisms for GRG and TPR. LG set penalties and incentives about their benefits and costs, i.e., if LG increase the amount of penalties, the benefits will increase; if the amount of incentives is reduced, the costs will be reduced. Higher administrative penalties increase the probability that LG will choose to supervise. In addition, the greater the probability of LG choosing to supervise, the greater the probability of TPR supervising the GRG, thus prompting the GRG to accelerate data security governance.

**Corollary 6** During the game’s evolution, the LG’s probability of supervising decreases as either the GRG opts to conduct data security governance, the TPR decides to supervise, or both.

**Proof:** When y<y**, i.e. y<y**, LG choose supervision as a stabilization strategy. As GRG choose data security governance and TPR choose regulation, the probability of LG choosing supervision decreases to 0. Thus the probability of LG supervision decreases as the probability of GRG choosing to conduct data security governance and TPR choosing to supervise increases.

**Corollary 6 shows that** the probability that a LG chooses to supervise is influenced by the TPR as well as the selection strategy of the GRG. When the probability of GRG choosing data security governance and TPR choosing to supervise increases, the probability of LG choosing to supervise decreases, making them prone to situations such as untimely supervision.

### Stability analysis

After analyzing the strategic stability of the GRG, the TPR, and the LG, we have reached the equilibrium point of their replicated dynamic equations. In other words, over time, the strategic choices of these critical players will converge to a state of equilibrium. However, while the final result of the above analysis indicates the equilibrium point where the equations converge, the stability of this final equilibrium point has yet to be determined. According to the evolutionary game theory, further analysis of the three-party game subjects is still necessary.

The replication dynamic equations for the three subjects, the GRG, the TPR, and the LG, are:


$$\mathrm{\backslash [}F(x)=\frac{dx}{dt}=x\left(E11-\overset{―}{E1}\right)=x(x-1)[E11-E12]\mathrm{\backslash ]}$$

$$=x(x-1[C_{z}-C_{b}-C_{p}-B_{j}-y(R_{p}-B_{j})-z(F_{p}+M_{p})]$$ (24)

$$\mathrm{\backslash [}F(y)=\frac{dy}{dt}=y\left(E21-\overset{―}{E2}\right)=y(y-1)[E21-E22]\mathrm{\backslash ]}$$

$$=y(y-1[(1-x)B_{j}-C_{j}-z(F_{j}+M_{j})]$$ (25)

$$\mathrm{\backslash [}F(z)=\frac{dz}{dt}=z\left(E31-\overset{―}{E3}\right)=x(x-1)[E31-E32]\mathrm{\backslash ]}$$

$$=z(z-1[x(F_{p}+M_{p}+T_{d})-xyT_{d}+C_{d}-F_{j}-F_{p}-T_{d}+y(F_{j}+M_{j}+T_{d})]$$ (26)

The Jacobian matrix for this dynamic equation can be expressed as:


$$\mathrm{\backslash [}J=[\begin{array}{ccc} \frac{\partial F(x)}{\partial x} & \frac{\partial F(x)}{\partial y} & \frac{\partial F(x)}{\partial z}\\ \frac{\partial F(y)}{\partial x} & \frac{\partial F(y)}{\partial y} & \frac{\partial F(y)}{\partial z}\\ \frac{\partial F(z)}{\partial x} & \frac{\partial F(z)}{\partial y} & \frac{\partial F(z)}{\partial z} \end{array}]\mathrm{\backslash ]}$$

$$\begin{array}{c} =[\begin{array}{ccc} (2x-1)\left[C_{z}-C_{b}-C_{p}-B_{j}-y\left(R_{p}-B_{j}\right)-z\left(F_{p}+M_{p}\right)\right] & x(x-1)\;[B_{j}-R_{p}] & x(x-1)\;[-F_{p}-M_{p}]\\ y(y-1)\;[-B_{j}] & \left(2y-1)\;[(1-x)B_{j}-C_{j}-z(F_{j}+M_{j})\right] & y(y-1)\;[-F_{j}-M_{j}\\ z(z-1)\;[F_{p}+M_{p}+T_{d}-yT_{d}] & z(z-1)\;[F_{j}+M_{j}+T_{d}-xT_{d}]\\ & \;\;\;\;\;\;\;\;\;\;\;\;\;\;\;\;\;\;\;\;\;\;\;\;\;\;\;\;\;\;\;\;\;\;\;\;\;\;\;\;\;\;\;\;\;\;\;\;\;\;\;\;\;\;\;\;\;\;\;\;\;\;\;\;\;\;\;\;\;\;\;\;\;\;\;\;\;\;\;\;\;\;\;\;\;\;\;\;\;\;\;\;\;\;\;\;\;\;\;\;\;\;\;\;\;\;\;\;\;\;\;\;\;\;\;\;\;\;\;\;\;\;\;\;\;\;\;\;\;\;\;\;\;\;\;\;\;\;(2z-1)\;[x(F_{p}+M_{p}+T_{d})-xyT_{d}+C_{d}-F_{j}-F_{p}-T_{d}+y(F_{j}+M_{j}+T_{d})] \end{array}] \end{array}$$

The Nash equilibrium can be obtained by making F(x)=0, F(y)=0, and F(z)=0 in the game system, i.e., E1(0,0,0), E2(0,0,1), E3(0,1,0), E4(0,1,1), E5(1,0,0), E6(1,0,1), E7(1,1,0), E8(1,1,1), $E9(0,\frac{T_{d}+F_{p}+F_{j}-C_{d}}{F_{j}+M_{j}+T_{d}},\frac{B_{j}-C_{j}}{F_{j}+M_{j}}$), $E10(\frac{T_{d}+F_{p}+F_{j}-C_{d}}{F_{p}+M_{p}+T_{d}},0,\frac{C_{z}-C_{b}-C_{p}-B_{j}}{F_{p}+M_{p}})$, $E11(\frac{B_{j}-C_{j}}{B_{j}},\frac{C_{z}-C_{b}-C_{p}-B_{j}}{R_{p}-B_{j}},0)$, $E12(1,\frac{F_{j}-M_{p}-C_{d}}{F_{j}+M_{j}},\frac{-C_{j}}{F_{j}+M_{j}})$, $E12(\frac{F_{p}-C_{d}-M_{j}}{F_{p}+M_{p}},1,\frac{C_{z}-C_{b}-C_{p}-R_{p}}{F_{p}+M_{p}})$, $E13(\frac{B_{j}-C_{j}-F_{j}-M_{j}}{B_{j}},\frac{C_{z}-C_{b}-C_{p}-B_{j}-F_{p}-M_{p}}{R_{p}-B_{j}},1)$.

In an asymmetric game, if the equilibrium E of the evolutionary game is an asymptotically stable state, it must be a strict Nash equilibrium. A strict Nash equilibrium, by definition, is a pure strategy equilibrium, and the equilibrium E is a pure strategy equilibrium [56]. Therefore, it is sufficient to discard E9-E13 and focus on analyzing the asymptotic stability of the pure strategy equilibrium points E1-E8. According to Lyapunov’s first theorem, the necessary condition for an equilibrium point to be locally asymptotically stable is that the real part of all eigenvalues of its corresponding Jacobian matrix must be negative [57]. In this paper, the stability of all pure strategy equilibrium points is determined through the eigenvalues of their Jacobian matrices, as shown in Table 3. When the real parts of all eigenvalues are negative (as in E2 and E7), the corresponding equilibrium is judged to be asymptotically stable (ESS). If there are eigenvalues with zero or positive real parts, the equilibrium point is unstable. Therefore, the results of the stability analysis are presented in Table 3.

**Table 3:** Equilibrium point stability analysis.

Balance Point	Jacobian matrix eigenvalue		Stability	Stable Condition
	λ1, λ2, λ3	Real Part Symbols		
E1(0,0.0)	$C_{j}-B_{j},$ $B_{j}+C_{b}+C_{p}-C_{z}$ $F_{j}-C_{d}+F_{p}+T_{d}$	−,−,+	Unstable	\
E2(0,0,1)	$C_{d}-F_{j}-F_{p}-T_{d}$, $C_{j}-B_{j}+F_{j}+M_{j}$ $B_{j}+C_{b}+C_{p}-C_{z}+F_{p}+M_{p}$	−,−,−	ESS	①
E3(0,1,0)	$B_{j}-C_{j}$, $F_{p}-C_{d}-M_{j}$, $C_{b}+C_{p}-C_{z}+R_{p}$	+,×,+	Unstable	\
E4(0,1,1)	$C_{d}-F_{p}+M_{j}$, $B_{j}-C_{j}-F_{j}-M_{j}$ $C_{b}+C_{p}-C_{z}+F_{p}+M_{p}+R_{p}$	×,+,+	Unstable	\
E5(1,0,0)	$C_{j}$, $F_{j}-C_{d}-M_{p}$, $C_{z}-C_{b}-C_{p}-B_{j}$	+,×,+	Unstable	\
E6(1,0,1)	$C_{j}+F_{j}+M_{j}$, $C_{d}-F_{j}+M_{p}$ $C_{z}-C_{b}-C_{p}-B_{j}-F_{p}-M_{p}$	+,×,+	Unstable	\
E7(1,1,0)	$-C_{j}$, ${-C}_{d}-M_{j}-M_{p}$, $C_{z}-C_{p}-C_{b}-R_{p}$	−,−,−	ESS	\
E8(1,1,1)	$C_{d}+M_{j}+M_{p}$, $-C_{j}$-$F_{j}$-$M_{j}$ $C_{z}-C_{p}-C_{b}-F_{p}-M_{p}-R_{p}$	+,−,−	Unstable	\

Note: × representation of inconclusive, ① $B_{j}+C_{b}+C_{p}-C_{z}+F_{p}+M_{p}<0$, $C_{j}-B_{j}+F_{j}+M_{j}<0$.

**Corollary 7** When $B_{j}+C_{b}+C_{p}-C_{z}+F_{p}+M_{p}<0$ and $C_{j}-B_{j}+F_{j}+M_{j}<0$, there exist two stable points E2(0,0,1) and E7(1,1,0).

**Proof:** When $B_{j}+C_{b}+C_{p}-C_{z}+F_{p}+M_{p}<0$ and $C_{j}-B_{j}+F_{j}+M_{j}<0$, it can be seen from Table 2 that the E2 and E7 eigenvalues are both negative, and at this time, there are two asymptotic stabilization points of the system.

**Corollary 7 shows that** When LG rewards and penalties for GRG and TPR are small, or when the cost of data governance for GRG is significant. At the same time, the cost of regulation for TPR is high, the choice of strategy stabilizes at strategy 1(GRG choose not to conduct data security governance, TPR choose not to supervise, and LG choose to supervise) and strategy 2(GRG choose to conduct data security governance, TPR choose to supervise, and LG choose not to supervise). To avoid the emergence of Strategy 1, LG should increase the penalties and incentives for TPR and GRG to fulfill the supervisory role of LG.

**Corollary 8** A stabilization point E7(1,1,0) exists when $F_{p}+M_{p}>C_{z}-C_{b}-C_{p}-B_{j}>$0 and $M_{j}+F_{j}>B_{j}-C_{j}>0$.

**Proof:** When $F_{p}+M_{p}>C_{z}-C_{b}-C_{p}-B_{j}>0$ and $M_{j}+F_{j}>B_{j}-C_{j}>0$, according to the equilibrium point stability analysis, condition j is not satisfied, E2 is an unstable point, and there is only one stable point as E7.

**Corollary 8 shows that** to effectively prevent the emergence of a strategy combination where x=0, y=0, z=1 in the evolutionary game model, the sum of rewards and penalties from the LG must exceed the difference between the cost of implementing data security governance and the cost of non-governance by the GRG, specifically, it must be higher than the cost of violations. This ensures the appearance of a mixed-strategy equilibrium point, provided that all other critical factors remain constant. Furthermore, when the net penalty (the difference between punishment and reward) exceeds the cost of regulation, it incentivizes GRG to adopt a data security governance strategy.

### Simulation experiments

#### Setting initial values.

Currently, there is a lack of comprehensive statistical data on the regulatory aspect of GRG data security governance. To ensure the validity and authenticity of the simulation data, we have set some parameters based on existing relevant data. These initial data primarily come from national standards, government websites, and other sources. The remaining parameters are set based on references from related literature, industry experts, and corporate data.

Upon reviewing the 2023 Statistical Yearbook of Xiangtan City, Hunan Province, particularly the fiscal expenditures section [58], we found that local governments allocated 46.03 million yuan for public safety expenditures. Since public safety expenditures cover a broad range, including data security governance as one of the components, we set the cost of GRG for data security governance, $C_{z}$ to 45. According to IBM’s “2023 Data Breach Cost Report” [59], the average cost of a data breach in 2023 reached 69.4 million yuan, which includes breach costs across more than 50 industries. Therefore, we set the cost for GRG not performing data security governance, $C_{b}$ to 15.

On June 16, 2023, a technology company in Zhejiang developed and operated an information management system for a local government department in Zhejiang, but failed to implement data security services, resulting in significant data leakage. The Wenzhou Public Security Bureau in Zhejiang imposed a fine of 1 million yuan on the company [60]. Based on this, we set the penalty value from LG to TPR, $F_{j}$ to 8. According to Article 45 of the “Data Security Law of the People’s Republic of China,” organizations that conduct data processing activities and fail to fulfill their data security protection obligations, resulting in significant data leakage or other serious consequences, are fined between 500,000 yuan and 2 million yuan. For violations of national core data management regulations that threaten national sovereignty, security, and development interests, relevant competent authorities impose fines ranging from 2 million yuan to 10 million yuan [61]. Therefore, we set the penalty from LG to GRG, $F_{p}$ to 15, and the penalty from the superior department to LG, $T_{d}$ to 40. On December 18, 2023, the Xicheng District of Beijing released the “Measures to Accelerate the High-Quality Development of the Data Element Market in Xicheng District (Draft for Comment)” [62], proposing rewards for those leading or participating in formulating data element-related standards for the country, Beijing, or specific industries. The rewards would be 1 million yuan, 400,000 yuan, and 200,000 yuan respectively for each item. Based on this, we set the reward from LG to GRG, $M_{p}$ to 7, and the reward from LG to TPR, $M_{j}$ to 4.

Other parameter values were set based on the previously described assumptions and stability conditions, as well as references from related literature [3,63,44]. The initial values of the parameters in Array 1 are shown in Table 4. These values are designed to satisfy the conditions $F_{p}+M_{p}>C_{z}-C_{b}-C_{p}-B_{j}>0$and$M_{j}+F_{j}>B_{j}-C_{j}>0$, and to analyze the impact of changing values on the evolutionary game process.

**Table 4:** Initial Parameter Settings.

Parameter	$R_{p}$	$C_{z}$	$C_{b}$	$C_{p}$	$B_{j}$	$F_{p}$	$M_{p}$	$C_{j}$	$F_{j}$	$M_{j}$	$C_{d}$	$T_{d}$
**Value**	40	45	15	10	20	15	7	10	8	4	8	40
**Unit**	Hundred Thousand											

## Results

### a. The effect of GRG raw benefits and TPR costs on the evolutionary game process

**Fig 5:**
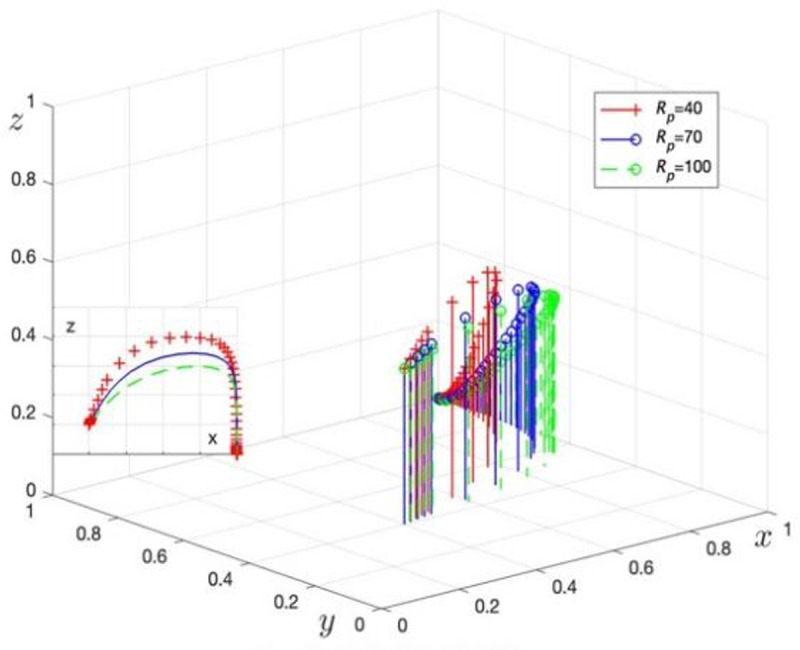
The impact of GRG’s original profit.

**Fig 6:**
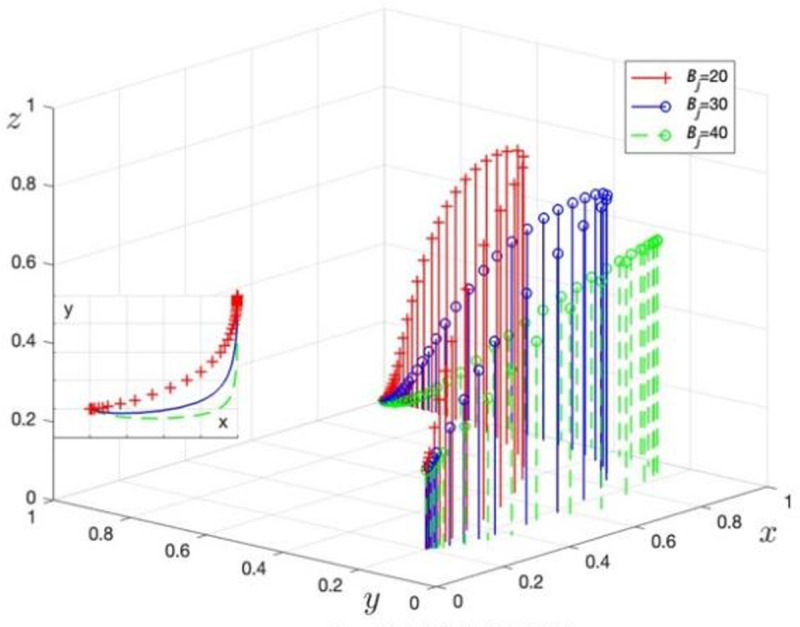
The impact of rent-seeking costs. b. The effect of LG fines and incentives for TPR on the evolutionary game process.

$R_{p}$ is assigned values of $R_{p}=$ 40, 70, and 100 to obtain three sets of control data, with the simulation results shown in Fig 5. $B_{j}$ is assigned values of $B_{j}=$ 20, 30, and 40 to obtain three sets of control data, with the simulation results shown in Fig 6.

Fig 5 illustrates the evolution of GRG strategy choices as the original revenue ($R_{p}$) changes. When $R_{p}$ increases from 40 to 100, the trend for GRG to choose data security governance accelerates. This result confirms Corollary 1, indicating that $R_{p}$ has a positive influence on GRG’s strategy choice.

Fig 6 shows the evolution of GRG strategy choices as rent-seeking costs ($B_{j}$) change. When $B_{j}$ increases from 20 to 40, the trend for GRG to choose data security governance accelerates, validating the effectiveness of Corollary 1 and demonstrating that an increase in $B_{j}$ positively affects GRG’s strategy choice.

**Fig 7:**
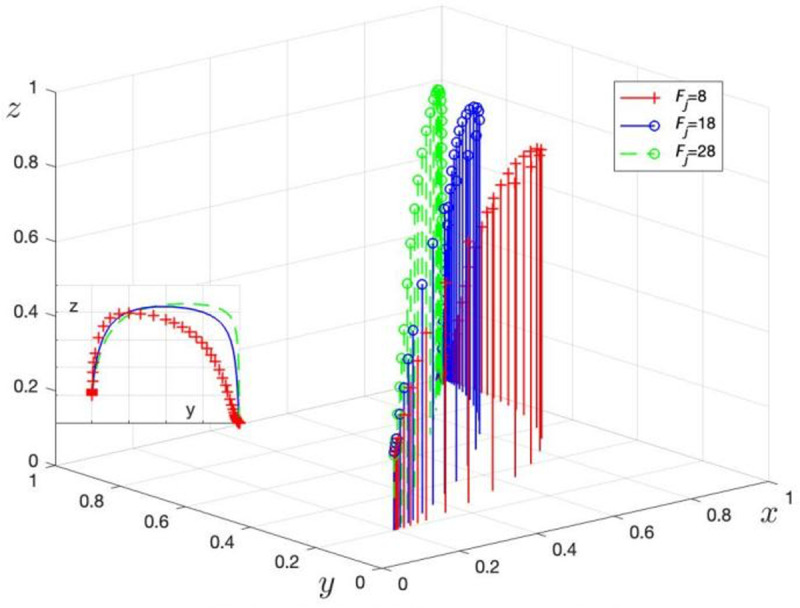
Effect of LG on the amount.

**Fig 8:**
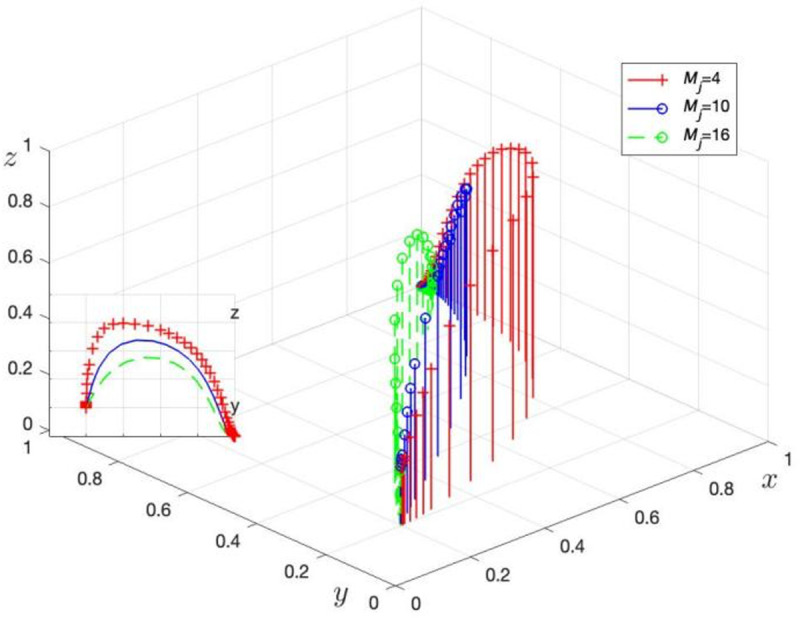
Effect of LG on the amount of fines imposed on TPR of incentives for TPR. c. The effect of LG incentives for GRG and administrative penalties at higher levels on the evolutionary game process.

$F_{j}$ is assigned values of $F_{j}=$ 8, 18, and 28 to obtain three sets of control data, with the simulation results shown in Fig 7. $M_{j}$ is assigned values of $M_{j}=$ 4, 10, and 16 to obtain three sets of control data, with the simulation results shown in Fig 8.

Fig 7 shows the evolution of LG strategy choices as the punishment level from TPR ($F_{j}$) changes. When $F_{j}$ increases from 8 to 28, the process for LG to choose regulation accelerates, in line with Corollary 5, indicating that an increase in $F_{j}$ positively guides LG towards choosing regulation. At the same time, the increase in $F_{j}$ raises the probability that TPR will choose not to supervise, avoiding rent-seeking behavior with GRG, consistent with the results of Corollary 3.

Fig 8 shows the evolution of LG strategy choices as the reward level from TPR ($M_{j}$) changes. When $M_{j}$ increases from 4 to 16, the process for LG to choose regulation accelerates. This simulation supports Corollary 5, indicating that an increase in $M_{j}$ encourages LG to choose non-regulation.

**Fig 9:**
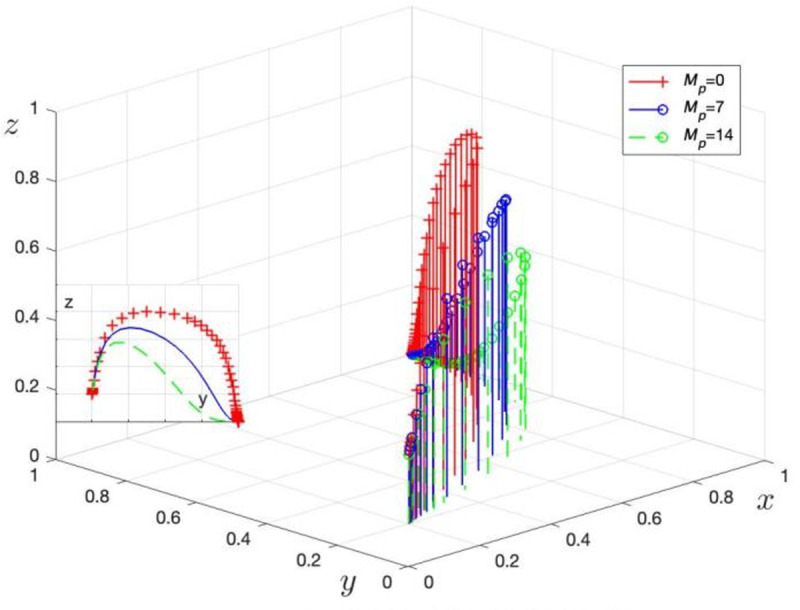
Effect of LG on the amount of incentives for GRG.

**Fig 10:**
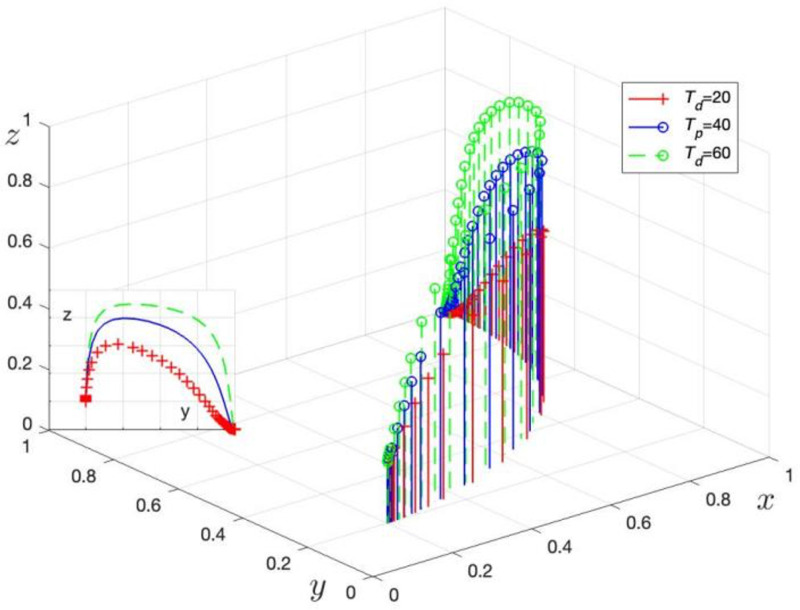
Effect of higher authorities on LG penalties.

$M_{p}$ is assigned values of $M_{p}=$ 0, 7, and 14 to obtain three sets of control data, with the simulation results shown in Fig 9. $T_{d}$ is assigned values of $T_{d}=$20, 40, and 60 to obtain three sets of control data, with the simulation results shown in Fig 10.

Fig 9 illustrates the evolution of LG strategy choices as the reward and punishment levels from GRG ($M_{p}$) change. When $M_{p}$ increases from 0 to 14, the process for LG to choose non-regulation accelerates. This result validates Corollary 5, showing that an increase in $M_{p}$ reduces the probability of LG choosing regulation.

Fig 10 demonstrates the simulation evolution of LG strategy choices as the upper-level punishment ($T_{d}$) changes. When $T_{d}$ increases from 20 to 60, the trend for LG to choose regulation accelerates. This result confirms Corollary 5, indicating that an increase in $T_{d}$ raises the probability of LG choosing regulation.

## Discussion

**Fig 11:**
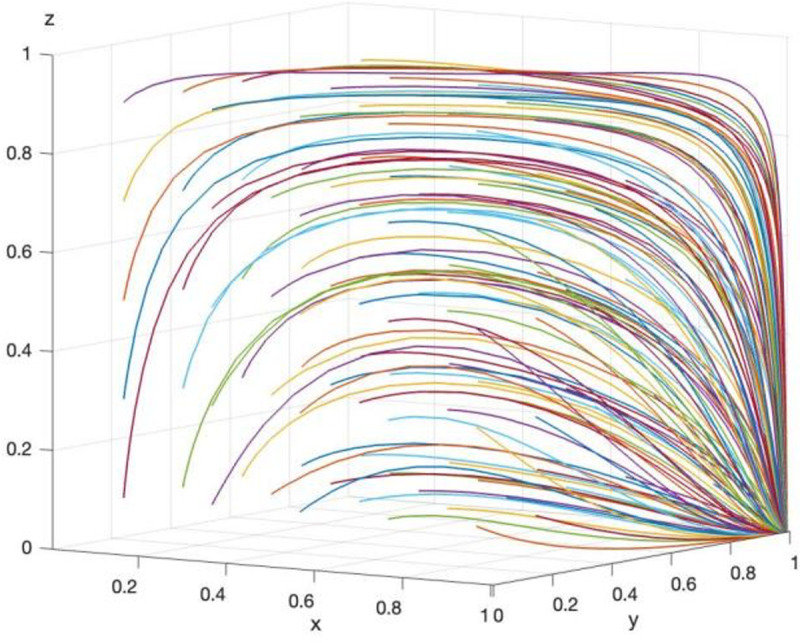
The result of evolving array 1 for 50 times.

**Fig 12:**
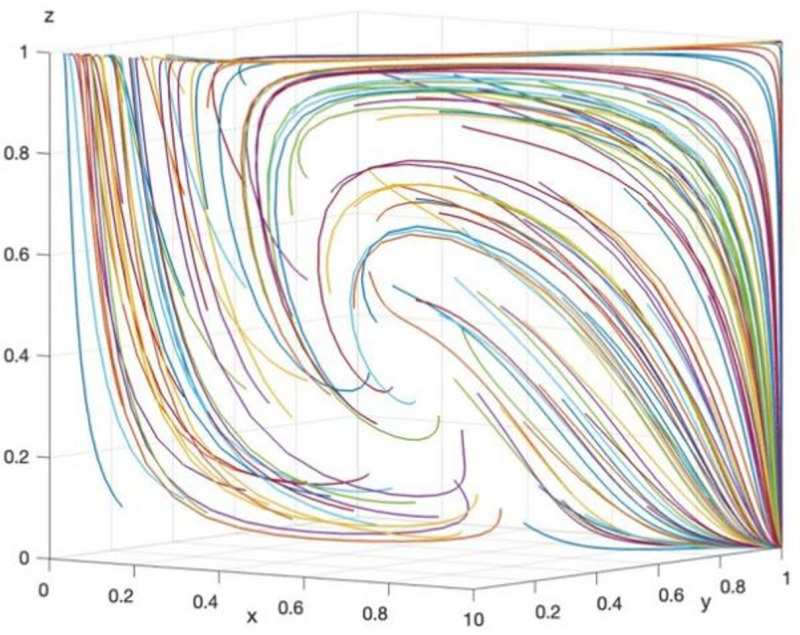
The result of evolving array 2 for 50 times.

Array 2 is set as follows: $R_{p}=40$, $C_{z}=45$, $C_{b}=5$, $C_{p}=5$, $B_{j}=25$, $F_{p}=7$, $M_{p}=3$, $C_{j}=8$, $F_{j}=6$, $M_{j}=2$, $C_{d}=8$, $T_{d}=15$. The parameter settings satisfy the conditions $B_{j}+C_{b}+C_{p}-C_{z}+F_{p}+M_{p}<0$ and $C_{j}-B_{j}+F_{j}+M_{j}<0$. The arrays 1 and 2 will be evolved for 50 times. The results are shown in Figs 11 and 12.

As shown in Fig 11, after 50 evolutionary analyses, the final strategy stabilizes at (1,1,0), which corresponds to “data security governance, supervision, no supervision,” in line with Corollary 8. According to Fig 12, when Array 2 satisfies ①, the final strategy stabilizes at both (0,0,1) and (1,1,0) after 50 evolutionary analyses. This indicates that the three stakeholders—GRG, TPR, and LG—form two strategy combinations: (no data security governance, no supervision, supervision) and (data security governance, supervision, no supervision). Evolutionary analysis suggests that LG should establish a reward and punishment mechanism based on the interaction between the choices of GRG and TPR, ensuring that the sum of fines and rewards exceeds the benefits derived from choosing not to supervise and engaging in rent-seeking behavior. This is critical to prevent TPR from opting out of supervision and engaging in rent-seeking behavior with GRG, which could threaten data security at the grassroots level. Therefore, the simulation analysis corroborates the inferred selection strategies of the involved parties and provides practical guidance for GRG’s data security supervision strategy.

## Conclusions

With the introduction of the reward and punishment mechanism, this study incorporates rent-seeking behavior into the construction of a three-party evolutionary game model to analyze the interactions between GRG, TPR, and LG in data security governance. Through theoretical analysis and model simulation, this study summarizes three core principles of cross-level governance: First, the reward mechanism should match the regulatory costs; excessive incentives may weaken the effectiveness of regulation. Second, the punishment measures should be sufficient to outweigh potential profit incentives, effectively curbing rent-seeking behavior. Finally, increasing the cost of rent-seeking is an effective method to prevent GRG from neglecting data security governance and TPR from failing to perform its regulatory duties, thus helping improve the overall compliance of the regulatory system.

By incorporating real-world cases, this study explores how dynamic reward and punishment mechanisms can be effectively integrated into the existing regulatory system to enhance regulatory effectiveness. For example, some LGs have implemented dynamic reward and punishment mechanisms in specific areas, such as carbon emissions [64] and drug safety [34]. In the carbon emissions field, LGs have used dynamic adjustments to reward mechanisms to encourage companies to reduce emissions, while adjusting penalty measures based on companies’ compliance, thus reducing environmental pollution and lowering regulatory costs. Additionally, in the field of drug safety, the government has increased the intensity of rewards and punishments for TPR to ensure drug quality and public health, preventing the entry of low-quality drugs into the market. Drawing from these practical experiences, this study proposes applying dynamic reward and punishment mechanisms to data security governance. LG can adjust rewards and punishments based on the compliance behavior of GRG and TPR, ensuring that all parties maintain a state beneficial to societal and public interests. Furthermore, by increasing the rent-seeking costs for GRG and TPR, the transparency and effectiveness of data governance can be enhanced, reducing regulatory gaps and lowering the risk of governance failure.

Although this study provides a new perspective on cross-level cooperation in data security governance, there are still some limitations. First, the assumptions in the model may deviate from the complexity of real-world environments, and future research could further validate the model’s adaptability using actual data. Second, this study mainly focuses on the digital governance context in China, and future research could consider cross-country comparative studies to explore the impact of different governance structures on data security. Finally, with the development of emerging technologies, future research could explore how to adjust regulatory frameworks to address the challenges posed by technological changes.

This work was supported by the Priority projects of the Social Science Achievement Review Board of HuNan: “A Study on the Governance Capacity of Rural Grassroots Governments in Hunan under the Perspective of ‘Digital Countryside’” [No. XSP24ZDI032].

This work was supported by the Priority projects of the Social Science Achievement Review Board of HuNan: “A Study on the Governance Capacity of Rural Grassroots Governments in Hunan under the Perspective of ‘Digital Countryside’” [No. XSP24ZDI032].

## Supporting information

S1 File This study uses MATLAB for simulation, and the following is the code used in this research. Through these codes, we can carry out the specific simulation process and conduct a detailed analysis of the topic under study. I hope this will assist your research, and I look forward to further communication and collaboration. (PDF)
