# Supplementary material for: Dynamic Tripartite Governance in data security: An evolutionary game model with cross-level government supervision
Source: PLoS One. 2025 Jun 3;20(6):e0325473. doi: 10.1371/journal.pone.0325473 (PMC12133017; doi:10.1371/journal.pone.0325473)
Supplement: S1 File — (PDF) [file pone.0325473.s001.pdf]

This study uses MATLAB for simulation, and the following is the code used in this research. Through these codes, we can carry out the specific simulation process and conduct a detailed analysis of the topic under study. I hope this will assist your research, and I look forward to further communication and collaboration.

```
%% 图5
clc,clear;
figure(5);
%Rp=40
Rp=40,Cz=45,Cb=15,Cp=10,Bj=20,Fp=15,Mp=7,Cj=10,Fj=8,Mj=4,Cd=8,Td=40;
[t,y]=ode45(@(t,y)
jianguan(t,y,Rp,Cz,Cb,Cp,Bj,Fp,Mp,Cj,Fj,Mj,Cd,Td),[0 50],[0.4 0.4
0.4]);
%plot3(y(:,1),y(:,2),y(:,3),'r+','linewidth',1);
stem3(y(:,1),y(:,2),y(:,3),'r+','linewidth',1); %三维火柴棍图
hold on ;
%Rp=70
Rp=70,Cz=45,Cb=15,Cp=10,Bj=20,Fp=15,Mp=7,Cj=10,Fj=8,Mj=4,Cd=8,Td=40;
[t,y]=ode45(@(t,y)
jianguan(t,y,Rp,Cz,Cb,Cp,Bj,Fp,Mp,Cj,Fj,Mj,Cd,Td),[0 50],[0.4 0.4
0.4]);
%plot3(y(:,1),y(:,2),y(:,3),'b-','linewidth',1);
stem3(y(:,1),y(:,2),y(:,3),'b-','linewidth',1);%三维火柴棍图
hold on  ;
%Rp=100
Rp=100,Cz=45,Cb=15,Cp=10,Bj=20,Fp=15,Mp=7,Cj=10,Fj=8,Mj=4,Cd=8,Td=40
;
[t,y]=ode45(@(t,y)
jianguan(t,y,Rp,Cz,Cb,Cp,Bj,Fp,Mp,Cj,Fj,Mj,Cd,Td),[0 50],[0.4 0.4
0.4]);
%plot3(y(:,1),y(:,2),y(:,3),'g--','linewidth',1);
stem3(y(:,1),y(:,2),y(:,3),'g--','linewidth',1);%三维火柴棍图
hold on  ;
%坐标刻度间隔及其区间，坐标标注
set(gca,'XTick',[0:0.2:1],'YTick',[0:0.2:1],'ZTick',[0:0.2:1])
axis([0 1 0 1 0 1])
xlabel('$x$','interpreter','latex');ylabel('$y$','interpreter','late
x');zlabel('$z$','interpreter','latex','Rotation',360,'position',
[-0.1 1 1.1]);
%图象网格，底图加白
grid on
hold on
set(0,'defaultfigurecolor','w')
%图例及标题
legend({'{\it\fontname{Bodoni MT}R_{p}}=40','{\it\fontname{Bodoni
MT}R_{p}}=70','{\it\fontname{Bodoni MT}R_{p}}
=100'},'location','northeast');
title('图  5 ','FontWeight','bold','position',[0 0 -0.2]);
% the small figure
axes('position',[0.13 0.32 0.2 0.2]); %小图的关键语句确定小图的大小位置
```

```
% z-x小图中的线条
%小图中的线条
Rp=40,Cz=45,Cb=15,Cp=10,Bj=20,Fp=15,Mp=7,Cj=10,Fj=8,Mj=4,Cd=8,Td=40;
[t,y]=ode45(@(t,y)
jianguan(t,y,Rp,Cz,Cb,Cp,Bj,Fp,Mp,Cj,Fj,Mj,Cd,Td),[0 50],[0.2 0.2
0.2]);
plot3(y(:,1),y(:,2),y(:,3),'r+','linewidth',1);
hold on
Rp=70,Cz=45,Cb=15,Cp=10,Bj=20,Fp=15,Mp=7,Cj=10,Fj=8,Mj=4,Cd=8,Td=40;
[t,y]=ode45(@(t,y)
jianguan(t,y,Rp,Cz,Cb,Cp,Bj,Fp,Mp,Cj,Fj,Mj,Cd,Td),[0 50],[0.2 0.2
0.2]);
plot3(y(:,1),y(:,2),y(:,3),'b-','linewidth',1);
hold on
Rp=100,Cz=45,Cb=15,Cp=10,Bj=20,Fp=15,Mp=7,Cj=10,Fj=8,Mj=4,Cd=8,Td=40
;
[t,y]=ode45(@(t,y)
jianguan(t,y,Rp,Cz,Cb,Cp,Bj,Fp,Mp,Cj,Fj,Mj,Cd,Td),[0 50],[0.2 0.2
0.2]);
plot3(y(:,1),y(:,2),y(:,3),'g--','linewidth',1);
hold on
%坐标刻度间隔及区间，但不显示
set(gca,'XTick',[0:0.2:1],'YTick',[0:0.2:1],'ZTick',[0:0.2:1])
axis([0 1 0 1 0 1])
set(gca,'XTickLabel','','YTickLabel','','ZTickLabel','')
%背景网格并设置白底
grid on
hold on
set(0,'defaultfigurecolor','w')
%图象显示视角，以及相应的坐标标注
view([0 0]);


%%% 图6
clc,clear;
figure(6);
%Bj=10
Rp=40,Cz=45,Cb=15,Cp=10,Bj=20,Fp=15,Mp=7,Cj=10,Fj=8,Mj=4,Cd=8,Td=40;
[t,y]=ode45(@(t,y)
jianguan(t,y,Rp,Cz,Cb,Cp,Bj,Fp,Mp,Cj,Fj,Mj,Cd,Td),[0 50],[0.2 0.2
0.2]);
%plot3(y(:,1),y(:,2),y(:,3),'r+','linewidth',1);
stem3(y(:,1),y(:,2),y(:,3),'r+','linewidth',1); %三维火柴棍图
hold on ;
%Bj=30
Rp=40,Cz=45,Cb=15,Cp=10,Bj=30,Fp=15,Mp=7,Cj=10,Fj=8,Mj=4,Cd=8,Td=40;
[t,y]=ode45(@(t,y)
jianguan(t,y,Rp,Cz,Cb,Cp,Bj,Fp,Mp,Cj,Fj,Mj,Cd,Td),[0 50],[0.2 0.2
0.2]);
%plot3(y(:,1),y(:,2),y(:,3),'b-','linewidth',1);
stem3(y(:,1),y(:,2),y(:,3),'b-','linewidth',1);%三维火柴棍图
hold on  ;
%Bj=40
```

```
Rp=40,Cz=45,Cb=15,Cp=10,Bj=40,Fp=15,Mp=7,Cj=10,Fj=8,Mj=4,Cd=8,Td=40;
[t,y]=ode45(@(t,y)
jianguan(t,y,Rp,Cz,Cb,Cp,Bj,Fp,Mp,Cj,Fj,Mj,Cd,Td),[0 50],[0.2 0.2
0.2]);
%plot3(y(:,1),y(:,2),y(:,3),'g--','linewidth',1);
stem3(y(:,1),y(:,2),y(:,3),'g--','linewidth',1);%三维火柴棍图
hold on  ;
%坐标刻度间隔及其区间，坐标标注
set(gca,'XTick',[0:0.2:1],'YTick',[0:0.2:1],'ZTick',[0:0.2:1])
axis([0 1 0 1 0 1])
xlabel('$x$','interpreter','latex');ylabel('$y$','interpreter','late
x');zlabel('$z$','interpreter','latex','Rotation',360,'position',
[-0.1 1 1.1]);
%图象网格，底图加白
grid on
hold on
set(0,'defaultfigurecolor','w')
%图例及标题
legend({'{\it\fontname{Bodoni MT}B_{j}}=20','{\it\fontname{Bodoni
MT}B_{j}}=30','{\it\fontname{Bodoni MT}B_{j}}
=40'},'location','northeast');
title('图  6 ','FontWeight','bold','position',[0 0 -0.2]);
% the small figure
axes('position',[0.13 0.32 0.2 0.2]); %小图的关键语句确定小图的大小位置
% y-x小图中的线条
%小图中的线条
Rp=40,Cz=45,Cb=15,Cp=10,Bj=20,Fp=15,Mp=7,Cj=10,Fj=8,Mj=4,Cd=8,Td=40;
[t,y]=ode45(@(t,y)
jianguan(t,y,Rp,Cz,Cb,Cp,Bj,Fp,Mp,Cj,Fj,Mj,Cd,Td),[0 50],[0.2 0.2
0.2]);
plot3(y(:,1),y(:,2),y(:,3),'r+','linewidth',1);
hold on
Rp=40,Cz=45,Cb=15,Cp=10,Bj=30,Fp=15,Mp=7,Cj=10,Fj=8,Mj=4,Cd=8,Td=40;
[t,y]=ode45(@(t,y)
jianguan(t,y,Rp,Cz,Cb,Cp,Bj,Fp,Mp,Cj,Fj,Mj,Cd,Td),[0 50],[0.2 0.2
0.2]);
plot3(y(:,1),y(:,2),y(:,3),'b-','linewidth',1);
hold on
Rp=40,Cz=45,Cb=15,Cp=10,Bj=40,Fp=15,Mp=7,Cj=10,Fj=8,Mj=4,Cd=8,Td=40;
[t,y]=ode45(@(t,y)
jianguan(t,y,Rp,Cz,Cb,Cp,Bj,Fp,Mp,Cj,Fj,Mj,Cd,Td),[0 50],[0.2 0.2
0.2]);
plot3(y(:,1),y(:,2),y(:,3),'g--','linewidth',1);
hold on
%坐标刻度间隔及区间，但不显示
set(gca,'XTick',[0:0.2:1],'YTick',[0:0.2:1],'ZTick',[0:0.2:1])
axis([0 1 0 1 0 1])
set(gca,'XTickLabel','','YTickLabel','','ZTickLabel','')
%背景网格并设置白底
grid on
hold on
set(0,'defaultfigurecolor','w')
```

```
%图象显示视角，以及相应的坐标标注
view([0 90]); %y-x小图
xlabel('x','position',[0.8 1 0.3])
ylabel('y','position',[0.1 1 0.8],'Rotation',360)-x小图
xlabel('x','position',[0.8 1 0.3])
zlabel('z','position',[0.1 1 0.8],'Rotation',360)


%% 图7
clc,clear;
figure(7);
%Fj=4
Rp=40,Cz=45,Cb=15,Cp=10,Bj=20,Fp=15,Mp=7,Cj=10,Fj=8,Mj=4,Cd=8,Td=40;
[t,y]=ode45(@(t,y)
jianguan(t,y,Rp,Cz,Cb,Cp,Bj,Fp,Mp,Cj,Fj,Mj,Cd,Td),[0 50],[0.2 0.2
0.2]);
%plot3(y(:,1),y(:,2),y(:,3),'r+','linewidth',1);
stem3(y(:,1),y(:,2),y(:,3),'r+','linewidth',1); %三维火柴棍图
hold on ;
%Fj=14
Rp=40,Cz=45,Cb=15,Cp=10,Bj=20,Fp=15,Mp=7,Cj=10,Fj=18,Mj=4,Cd=8,Td=40
;
[t,y]=ode45(@(t,y)
jianguan(t,y,Rp,Cz,Cb,Cp,Bj,Fp,Mp,Cj,Fj,Mj,Cd,Td),[0 50],[0.2 0.2
0.2]);
%plot3(y(:,1),y(:,2),y(:,3),'b-','linewidth',1);
stem3(y(:,1),y(:,2),y(:,3),'b-','linewidth',1); %三维火柴棍图
hold on  ;
%Fj=24
Rp=40,Cz=45,Cb=15,Cp=10,Bj=20,Fp=15,Mp=7,Cj=10,Fj=28,Mj=4,Cd=8,Td=40
;
[t,y]=ode45(@(t,y)
jianguan(t,y,Rp,Cz,Cb,Cp,Bj,Fp,Mp,Cj,Fj,Mj,Cd,Td),[0 50],[0.2 0.2
0.2]);
%plot3(y(:,1),y(:,2),y(:,3),'g--','linewidth',1);
stem3(y(:,1),y(:,2),y(:,3),'g--','linewidth',1); %三维火柴棍图
hold on  ;
%坐标刻度间隔及其区间，坐标标注
set(gca,'XTick',[0:0.2:1],'YTick',[0:0.2:1],'ZTick',[0:0.2:1])
axis([0 1 0 1 0 1])
xlabel('$x$','interpreter','latex');ylabel('$y$','interpreter','late
x');zlabel('$z$','interpreter','latex','Rotation',360,'position',
[-0.1 1 1.1]);
%图象网格，底图加白
grid on
hold on
set(0,'defaultfigurecolor','w')
%图例及标题
legend({'{\it\fontname{Bodoni MT}F_{j}}=8','{\it\fontname{Bodoni MT}
F_{j}}=18','{\it\fontname{Bodoni MT}F_{j}}
=28'},'location','northeast');
title('图  7 ','FontWeight','bold','position',[0 0 -0.2]);
```

```
% the small figure
axes('position',[0.13 0.32 0.2 0.2]); %小图的关键语句确定小图的大小位置
% y-z小图中的线条
%小图中的线条
Rp=40,Cz=45,Cb=15,Cp=10,Bj=20,Fp=15,Mp=7,Cj=10,Fj=8,Mj=4,Cd=8,Td=40;
[t,y]=ode45(@(t,y)
jianguan(t,y,Rp,Cz,Cb,Cp,Bj,Fp,Mp,Cj,Fj,Mj,Cd,Td),[0 50],[0.2 0.2
0.2]);
plot3(y(:,1),y(:,2),y(:,3),'r+','linewidth',1);
hold on
Rp=40,Cz=45,Cb=15,Cp=10,Bj=20,Fp=15,Mp=7,Cj=10,Fj=18,Mj=4,Cd=8,Td=40
;
[t,y]=ode45(@(t,y)
jianguan(t,y,Rp,Cz,Cb,Cp,Bj,Fp,Mp,Cj,Fj,Mj,Cd,Td),[0 50],[0.2 0.2
0.2]);
plot3(y(:,1),y(:,2),y(:,3),'b-','linewidth',1);
hold on
Rp=40,Cz=45,Cb=15,Cp=10,Bj=20,Fp=15,Mp=7,Cj=10,Fj=28,Mj=4,Cd=8,Td=40
;
[t,y]=ode45(@(t,y)
jianguan(t,y,Rp,Cz,Cb,Cp,Bj,Fp,Mp,Cj,Fj,Mj,Cd,Td),[0 50],[0.2 0.2
0.2]);
plot3(y(:,1),y(:,2),y(:,3),'g--','linewidth',1);
hold on
%坐标刻度间隔及区间，但不显示
set(gca,'XTick',[0:0.2:1],'YTick',[0:0.2:1],'ZTick',[0:0.2:1])
axis([0 1 0 1 0 1])
set(gca,'XTickLabel','','YTickLabel','','ZTickLabel','')
%背景网格并设置白底
grid on
hold on
set(0,'defaultfigurecolor','w')
%图象显示视角，以及相应的坐标标注
view([90 0]); %y-z小图
ylabel('y','position',[0.8 1 0.3])
zlabel('z','position',[0.1 1 0.8],'Rotation',360)


%% 图8
clc,clear;
figure(8);
%Mj=4
Rp=40,Cz=45,Cb=15,Cp=10,Bj=20,Fp=15,Mp=7,Cj=10,Fj=8,Mj=4,Cd=8,Td=40;
[t,y]=ode45(@(t,y)
jianguan(t,y,Rp,Cz,Cb,Cp,Bj,Fp,Mp,Cj,Fj,Mj,Cd,Td),[0 50],[0.2 0.2
0.2]);
%plot3(y(:,1),y(:,2),y(:,3),'r+','linewidth',1);
stem3(y(:,1),y(:,2),y(:,3),'r+','linewidth',1); %三维火柴棍图
hold on ;
%Mj=10
Rp=40,Cz=45,Cb=15,Cp=10,Bj=20,Fp=15,Mp=7,Cj=10,Fj=8,Mj=10,Cd=8,Td=40
;
```

```
[t,y]=ode45(@(t,y)
jianguan(t,y,Rp,Cz,Cb,Cp,Bj,Fp,Mp,Cj,Fj,Mj,Cd,Td),[0 50],[0.2 0.2
0.2]);
%plot3(y(:,1),y(:,2),y(:,3),'b-','linewidth',1);
stem3(y(:,1),y(:,2),y(:,3),'b-','linewidth',1); %三维火柴棍图
hold on  ;
%Mj=16
Rp=40,Cz=45,Cb=15,Cp=10,Bj=20,Fp=15,Mp=7,Cj=10,Fj=8,Mj=16,Cd=8,Td=40
;
[t,y]=ode45(@(t,y)
jianguan(t,y,Rp,Cz,Cb,Cp,Bj,Fp,Mp,Cj,Fj,Mj,Cd,Td),[0 50],[0.2 0.2
0.2]);
%plot3(y(:,1),y(:,2),y(:,3),'g--','linewidth',1);
stem3(y(:,1),y(:,2),y(:,3),'g--','linewidth',1); %三维火柴棍图
hold on  ;
%坐标刻度间隔及其区间，坐标标注
set(gca,'XTick',[0:0.2:1],'YTick',[0:0.2:1],'ZTick',[0:0.2:1])
axis([0 1 0 1 0 1])
xlabel('$x$','interpreter','latex');ylabel('$y$','interpreter','late
x');zlabel('$z$','interpreter','latex','Rotation',360,'position',
[-0.1 1 1.1]);
%图象网格，底图加白
grid on
hold on
set(0,'defaultfigurecolor','w')
%图例及标题
legend({'{\it\fontname{Bodoni MJ}M_{j}}=4','{\it\fontname{Bodoni MJ}
M_{j}}=10','{\it\fontname{Bodoni MJ}M_{j}}
=16'},'location','northeast');
title('图  8 ','FontWeight','bold','position',[0 0 -0.2]);
% the small figure
axes('position',[0.13 0.32 0.2 0.2]); %小图的关键语句确定小图的大小位置
% y-z小图中的线条
%小图中的线条
Rp=40,Cz=45,Cb=15,Cp=10,Bj=20,Fp=15,Mp=7,Cj=10,Fj=8,Mj=4,Cd=8,Td=40;
[t,y]=ode45(@(t,y)
jianguan(t,y,Rp,Cz,Cb,Cp,Bj,Fp,Mp,Cj,Fj,Mj,Cd,Td),[0 50],[0.2 0.2
0.2]);
plot3(y(:,1),y(:,2),y(:,3),'r+','linewidth',1);
hold on
Rp=40,Cz=45,Cb=15,Cp=10,Bj=20,Fp=15,Mp=7,Cj=10,Fj=8,Mj=10,Cd=8,Td=40
;
[t,y]=ode45(@(t,y)
jianguan(t,y,Rp,Cz,Cb,Cp,Bj,Fp,Mp,Cj,Fj,Mj,Cd,Td),[0 50],[0.2 0.2
0.2]);
plot3(y(:,1),y(:,2),y(:,3),'b-','linewidth',1);
hold on
Rp=40,Cz=45,Cb=15,Cp=10,Bj=20,Fp=15,Mp=7,Cj=10,Fj=8,Mj=16,Cd=8,Td=40
;
[t,y]=ode45(@(t,y)
jianguan(t,y,Rp,Cz,Cb,Cp,Bj,Fp,Mp,Cj,Fj,Mj,Cd,Td),[0 50],[0.2 0.2
0.2]);
```

```
plot3(y(:,1),y(:,2),y(:,3),'g--','linewidth',1);
hold on
%坐标刻度间隔及区间，但不显示
set(gca,'XTick',[0:0.2:1],'YTick',[0:0.2:1],'ZTick',[0:0.2:1])
axis([0 1 0 1 0 1])
set(gca,'XTickLabel','','YTickLabel','','ZTickLabel','')
%背景网格并设置白底
grid on
hold on
set(0,'defaultfigurecolor','w')
%图象显示视角，以及相应的坐标标注
view([90 0]); %y-z小图
ylabel('y','position',[0.8 1 0.3])
zlabel('z','position',[0.1 1 0.8],'Rotation',360)


%% 图9
clc,clear;
figure(9);
%Mp=0
Rp=40,Cz=45,Cb=15,Cp=10,Bj=20,Fp=15,Mp=0,Cj=10,Fj=8,Mj=4,Cd=8,Td=40;
[t,y]=ode45(@(t,y)
jianguan(t,y,Rp,Cz,Cb,Cp,Bj,Fp,Mp,Cj,Fj,Mj,Cd,Td),[0 50],[0.2 0.2
0.2]);
%plot3(y(:,1),y(:,2),y(:,3),'r+','linewidth',1);
stem3(y(:,1),y(:,2),y(:,3),'r+','linewidth',1); %三维火柴棍图
hold on ;
%Mp=7
Rp=40,Cz=45,Cb=15,Cp=10,Bj=20,Fp=15,Mp=7,Cj=10,Fj=8,Mj=4,Cd=8,Td=40;
[t,y]=ode45(@(t,y)
jianguan(t,y,Rp,Cz,Cb,Cp,Bj,Fp,Mp,Cj,Fj,Mj,Cd,Td),[0 50],[0.2 0.2
0.2]);
%plot3(y(:,1),y(:,2),y(:,3),'b-','linewidth',1);
stem3(y(:,1),y(:,2),y(:,3),'b-','linewidth',1); %三维火柴棍图
hold on  ;
%Mp=14
Rp=40,Cz=45,Cb=15,Cp=10,Bj=20,Fp=15,Mp=14,Cj=10,Fj=8,Mj=4,Cd=8,Td=40
;
[t,y]=ode45(@(t,y)
jianguan(t,y,Rp,Cz,Cb,Cp,Bj,Fp,Mp,Cj,Fj,Mj,Cd,Td),[0 50],[0.2 0.2
0.2]);
%plot3(y(:,1),y(:,2),y(:,3),'g--','linewidth',1);
stem3(y(:,1),y(:,2),y(:,3),'g--','linewidth',1); %三维火柴棍图
hold on  ;
%坐标刻度间隔及其区间，坐标标注
set(gca,'XTick',[0:0.2:1],'YTick',[0:0.2:1],'ZTick',[0:0.2:1])
axis([0 1 0 1 0 1])
xlabel('$x$','interpreter','latex');ylabel('$y$','interpreter','late
x');zlabel('$z$','interpreter','latex','Rotation',360,'position',
[-0.1 1 1.1]);
%图象网格，底图加白
grid on
```

```
hold on
set(0,'defaultfigurecolor','w')
%图例及标题
legend({'{\it\fontname{Bodoni MT}M_{p}}=0','{\it\fontname{Bodoni MT}M_{p}}=7','{\it\fontname{Bodoni MT}M_{p}}=14'},'location','northeast');
title('图  9 ','FontWeight','bold','position',[0 0 -0.2]);
% the small figure
axes('position',[0.13 0.32 0.2 0.2]); %小图的关键语句确定小图的大小位置
% y-z小图中的线条
%小图中的线条
Rp=40,Cz=45,Cb=15,Cp=10,Bj=20,Fp=15,Mp=0,Cj=10,Fj=8,Mj=4,Cd=8,Td=40;
[t,y]=ode45(@(t,y) jianguan(t,y,Rp,Cz,Cb,Cp,Bj,Fp,Mp,Cj,Fj,Mj,Cd,Td),[0 50],[0.2 0.2 0.2]);
plot3(y(:,1),y(:,2),y(:,3),'r+','linewidth',1);
hold on
Rp=40,Cz=45,Cb=15,Cp=10,Bj=20,Fp=15,Mp=7,Cj=10,Fj=8,Mj=4,Cd=8,Td=40;
[t,y]=ode45(@(t,y) jianguan(t,y,Rp,Cz,Cb,Cp,Bj,Fp,Mp,Cj,Fj,Mj,Cd,Td),[0 50],[0.2 0.2 0.2]);
plot3(y(:,1),y(:,2),y(:,3),'b-','linewidth',1);
hold on
Rp=40,Cz=45,Cb=15,Cp=10,Bj=20,Fp=15,Mp=14,Cj=10,Fj=8,Mj=4,Cd=8,Td=40;
[t,y]=ode45(@(t,y) jianguan(t,y,Rp,Cz,Cb,Cp,Bj,Fp,Mp,Cj,Fj,Mj,Cd,Td),[0 50],[0.2 0.2 0.2]);
plot3(y(:,1),y(:,2),y(:,3),'g--','linewidth',1);
hold on
%坐标刻度间隔及区间，但不显示
set(gca,'XTick',[0:0.2:1],'YTick',[0:0.2:1],'ZTick',[0:0.2:1])
axis([0 1 0 1 0 1])
set(gca,'XTickLabel','','YTickLabel','','ZTickLabel','')
%背景网格并设置白底
grid on
hold on
set(0,'defaultfigurecolor','w')
%图象显示视角，以及相应的坐标标注
view([90 0]); %y-z小图
ylabel('y','position',[0.8 0.5 0.3])
zlabel('z','position',[0.75 0.5 0.5],'Rotation',360)


%% 图10
clc,clear;
figure(10);
%Td=20
Rp=40,Cz=45,Cb=15,Cp=10,Bj=20,Fp=15,Mp=7,Cj=10,Fj=8,Mj=4,Cd=8,Td=20;
[t,y]=ode45(@(t,y) jianguan(t,y,Rp,Cz,Cb,Cp,Bj,Fp,Mp,Cj,Fj,Mj,Cd,Td),[0 50],[0.2 0.2 0.2]);
```

```
%plot3(y(:,1),y(:,2),y(:,3),'r+','linewidth',1);
stem3(y(:,1),y(:,2),y(:,3),'r+','linewidth',1); %三维火柴棍图
hold on ;
%Td=40
Rp=40,Cz=45,Cb=15,Cp=10,Bj=20,Fp=15,Mp=7,Cj=10,Fj=8,Mj=4,Cd=8,Td=40;
[t,y]=ode45(@(t,y)
jianguan(t,y,Rp,Cz,Cb,Cp,Bj,Fp,Mp,Cj,Fj,Mj,Cd,Td),[0 50],[0.2 0.2
0.2]);
%plot3(y(:,1),y(:,2),y(:,3),'b-','linewidth',1);
stem3(y(:,1),y(:,2),y(:,3),'b-','linewidth',1); %三维火柴棍图
hold on  ;
%Td=60
Rp=40,Cz=45,Cb=15,Cp=10,Bj=20,Fp=15,Mp=7,Cj=10,Fj=8,Mj=4,Cd=8,Td=60;
[t,y]=ode45(@(t,y)
jianguan(t,y,Rp,Cz,Cb,Cp,Bj,Fp,Mp,Cj,Fj,Mj,Cd,Td),[0 50],[0.2 0.2
0.2]);
%plot3(y(:,1),y(:,2),y(:,3),'g--','linewidth',1);
stem3(y(:,1),y(:,2),y(:,3),'g--','linewidth',1); %三维火柴棍图
hold on  ;
%坐标刻度间隔及其区间，坐标标注
set(gca,'XTick',[0:0.2:1],'YTick',[0:0.2:1],'ZTick',[0:0.2:1])
axis([0 1 0 1 0 1])
xlabel('$x$','interpreter','latex');ylabel('$y$','interpreter','late
x');zlabel('$z$','interpreter','latex','Rotation',360,'position',
[-0.1 1 1.1]);
%图象网格，底图加白
grid on
hold on
set(0,'defaultfigurecolor','w')
%图例及标题
legend({'{\it\fontname{Bodoni MT}T_{d}}=20','{\it\fontname{Bodoni
MT}T_{p}}=40','{\it\fontname{Bodoni MT}T_{d}}
=60'},'location','northeast');
title('图  10 ','FontWeight','bold','position',[0 0 -0.2]);
% the small figure
axes('position',[0.13 0.32 0.2 0.2]); %小图的关键语句确定小图的大小位置
% y-z小图中的线条
%小图中的线条
Rp=40,Cz=45,Cb=15,Cp=10,Bj=20,Fp=15,Mp=7,Cj=10,Fj=8,Mj=4,Cd=8,Td=20;
[t,y]=ode45(@(t,y)
jianguan(t,y,Rp,Cz,Cb,Cp,Bj,Fp,Mp,Cj,Fj,Mj,Cd,Td),[0 50],[0.2 0.2
0.2]);
plot3(y(:,1),y(:,2),y(:,3),'r+','linewidth',1);
hold on
Rp=40,Cz=45,Cb=15,Cp=10,Bj=20,Fp=15,Mp=7,Cj=10,Fj=8,Mj=4,Cd=8,Td=40;
[t,y]=ode45(@(t,y)
jianguan(t,y,Rp,Cz,Cb,Cp,Bj,Fp,Mp,Cj,Fj,Mj,Cd,Td),[0 50],[0.2 0.2
0.2]);
plot3(y(:,1),y(:,2),y(:,3),'b-','linewidth',1);
hold on
Rp=40,Cz=45,Cb=15,Cp=10,Bj=20,Fp=15,Mp=7,Cj=10,Fj=8,Mj=4,Cd=8,Td=60;
[t,y]=ode45(@(t,y)
```

```
jianguan(t,y,Rp,Cz,Cb,Cp,Bj,Fp,Mp,Cj,Fj,Mj,Cd,Td),[0 50],[0.2 0.2
0.2]);
plot3(y(:,1),y(:,2),y(:,3),'g--','linewidth',1);
hold on
%坐标刻度间隔及区间，但不显示
set(gca,'XTick',[0:0.2:1],'YTick',[0:0.2:1],'ZTick',[0:0.2:1])
axis([0 1 0 1 0 1])
set(gca,'XTickLabel','','YTickLabel','','ZTickLabel','')
%背景网格并设置白底
grid on
hold on
set(0,'defaultfigurecolor','w')
%图象显示视角，以及相应的坐标标注
view([90 0]); %y-z小图
ylabel('y','position',[0.8 1 0.3])
zlabel('z','position',[0.75 0.5 0.5],'Rotation',360)


%%
%图11，数组1
clc,clear;
figure(11);
Rp=150,Cz=45,Cb=15,Cp=10,Bj=20,Fp=15,Mp=7,Cj=10,Fj=8,Mj=4,Cd=8,Td=15
;
for i=0.1:0.2:1
    for j=0.1:0.2:1
        for k=0.1:0.2:1
        [t,y]=ode45(@(t,y)
jianguan(t,y,Rp,Cz,Cb,Cp,Bj,Fp,Mp,Cj,Fj,Mj,Cd,Td),[0 50],[i j k]);
        plot3(y(:,1),y(:,2),y(:,3),'linewidth',1);
        set(gca,'XTick',[0:0.2:1],'YTick',[0:0.2:1],'ZTick',
[0:0.2:1])
        hold on
        axis([0 1 0 1 0 1])
        view([45 10])
        end
    end
end
grid on
hold on
xlabel('x','Rotation',0);
ylabel('y','Rotation',0);
zlabel('z','Rotation',360,'position',[0 0 1.05]);
title('图  11 数组1演化50次结果','FontWeight','bold','position',[1 0
-0.13]);


%%
%图12，数组2
clc,clear;
figure(12);
Rp=40,Cz=45,Cb=5,Cp=5,Bj=25,Fp=7,Mp=3,Cj=8,Fj=6,Mj=2,Cd=8,Td=15;
for i=0.1:0.2:1
```

```
    for j=0.1:0.2:1
        for k=0.1:0.2:1
        [t,y]=ode45(@(t,y)
jianguan(t,y,Rp,Cz,Cb,Cp,Bj,Fp,Mp,Cj,Fj,Mj,Cd,Td),[0 50],[i j k]);
        plot3(y(:,1),y(:,2),y(:,3),'linewidth',1);
        set(gca,'XTick',[0:0.2:1],'YTick',[0:0.2:1],'ZTick',
[0:0.2:1])
        hold on
        axis([0 1 0 1 0 1])
        view([45 10])
        end
    end
end
grid on
hold on
xlabel('x','Rotation',0);
ylabel('y','Rotation',0);
zlabel('z','Rotation',360,'position',[0 0 1.05]);
title('图  12 数组2演化50次结果','FontWeight','bold','position',[1 0
-0.13]);
```
